# Supplementary material for: Deletion of wfs1 Impairs Oligodendrocyte Precursor Cells Dorsal Distribution and Myelination Through the wfs1-hmgcs1 Axis in Zebrafish
Source: Biology (Basel). 2026 Aug 16;15(16):1402. doi: 10.3390/biology15161402 (PMC13510151; doi:10.3390/biology15161402)
Supplement: Supplementary file 1 [file biology-15-01402-s001.zip › File S1-Transcriptome sequencing data and differentially expressed genes (DEGs) in wfs1-- zebrafish at 5 dpf/2.KEGG/wfs1KOvsWT/ALL/plot/wfs1KOvsWT.html]

### wfs1KOvsWT KEGG通路富集列表

---

```
## Warning: package 'knitr' was built under R version 3.5.1
```

| KEGGID | Description | GeneRatio | BgRatio | pvalue | padj | geneID | geneName | keggID | Count | Up | Up\_Gene\_id | Down | Down\_Gene\_id |
| --- | --- | --- | --- | --- | --- | --- | --- | --- | --- | --- | --- | --- | --- |
| dre00590 | Arachidonic acid metabolism | 18/900 | 72/8379 | 0.0004558 | 0.0430928 | 406449\_1/406449/494108/767759/559020/406370\_1/494108\_1/393297/406370/566996/406575\_1/393592/797309/566996\_1/560649/399485/767688/100148818 | ptges3a/ptges3a/ptgr1/zgc:153704/ptgs2b/pla2g6/ptgr1/zgc:56585/pla2g6/si:dkey-11k2.7/lta4h/cyp2p9/cyp2j20/si:dkey-11k2.7/si:ch211-214p16.3/cyp2p10/zgc:154040/si:ch73-55i23.1 | dre:406449/dre:406449/dre:494108/dre:767759/dre:559020/dre:406370/dre:494108/dre:393297/dre:406370/dre:566996/dre:406575/dre:393592/dre:797309/dre:566996/dre:560649/dre:399485/dre:767688/dre:100148818 | 18 | 9 | 406449\_1/494108/559020/393297/406370/566996/406575\_1/393592/560649 | 9 | 406449/767759/406370\_1/494108\_1/797309/566996\_1/399485/767688/100148818 |
| dre00270 | Cysteine and methionine metabolism | 18/900 | 73/8379 | 0.0005455 | 0.0430928 | 335715/334195\_1/103911233/317744\_1/550524\_1/571201/561410\_1/791167/431773/445818/406688/431773\_1/30659\_1/103911241/563642/450084\_1/334195/678634 | mdh1ab/mat1a/LOC103911233/dnmt3bb.1/cbsa/si:ch73-97h19.2/tat/zgc:158222/enoph1/cthl/got2a/enoph1/dnmt3bb.2/LOC103911241/sdsl/gss/mat1a/mat2al | dre:335715/dre:334195/dre:103911233/dre:317744/dre:550524/dre:571201/dre:561410/dre:791167/dre:431773/dre:445818/dre:406688/dre:431773/dre:30659/dre:571201/dre:563642/dre:450084/dre:334195/dre:678634 | 18 | 11 | 335715/334195\_1/103911233/317744\_1/561410\_1/445818/431773\_1/30659\_1/103911241/563642/450084\_1 | 7 | 550524\_1/571201/791167/431773/406688/334195/678634 |
| dre04672 | Intestinal immune network for IgA production | 11/900 | 37/8379 | 0.0012708 | 0.0562322 | 799354/799354\_1/30762/368878/100006078\_1/30763/571282/100006078/797491\_1/100151365\_1/100333914 | cxcl12b/cxcl12b/mhc2dab/si:busm1-266f07.2/ccr9b/mhc2dcb/LOC571282/ccr9b/itgb7/si:dkey-1h24.6/ccl25a | dre:799354/dre:799354/dre:30762/dre:368878/dre:100006078/dre:30763/dre:571282/dre:100006078/dre:797491/dre:100151365/dre:100333914 | 11 | 8 | 799354/30762/368878/30763/571282/100006078/100151365\_1/100333914 | 3 | 799354\_1/100006078\_1/797491\_1 |
| dre01200 | Carbon metabolism | 32/900 | 173/8379 | 0.0014236 | 0.0562322 | 335715/560753\_1/560753/116517/568001\_2/445233/322670\_1/550579/368262/560827\_1/406762\_1/403070\_1/437019\_1/541329/570579\_1/407673/100006589\_1/541329\_1/550579\_1/403070/561416\_1/792824\_1/317746/406339\_1/561416/406688/393427/406702/563642/321224/751668/568001\_3 | mdh1ab/tpi1b/tpi1b/adh5/pfkmb/me2/aco2/idh3g/dlst/prps1b/pgd/rgn/pcca/gcsha/g6pd/rpia/idh1/gcsha/idh3g/rgn/pfkpb/pgp/suclg2/hk2/pfkpb/got2a/zgc:63568/pdha1a/sdsl/hkdc1/gck/pfkmb | dre:335715/dre:560753/dre:560753/dre:116517/dre:568001/dre:445233/dre:322670/dre:550579/dre:368262/dre:560827/dre:406762/dre:403070/dre:437019/dre:541329/dre:570579/dre:407673/dre:100006589/dre:541329/dre:550579/dre:403070/dre:561416/dre:792824/dre:317746/dre:406339/dre:561416/dre:406688/dre:393427/dre:406702/dre:563642/dre:321224/dre:751668/dre:568001 | 32 | 19 | 335715/560753\_1/116517/445233/560827\_1/406762\_1/403070\_1/437019\_1/541329/570579\_1/100006589\_1/550579\_1/561416\_1/317746/406702/563642/321224/751668/568001\_3 | 13 | 560753/568001\_2/322670\_1/550579/368262/407673/541329\_1/403070/792824\_1/406339\_1/561416/406688/393427 |
| dre00010 | Glycolysis / Gluconeogenesis | 20/900 | 103/8379 | 0.0060429 | 0.1717395 | 560753\_1/560753/100005587\_1/116517/100536795/568001\_2/406473/368239/561416\_1/406339\_1/561416/393427/406702/445326\_1/445326/321224/436646/751668/100006238\_1/568001\_3 | tpi1b/tpi1b/aldh9a1a.1/adh5/wu:fb14d09/pfkmb/pck2/aldh2.2/pfkpb/hk2/pfkpb/zgc:63568/pdha1a/akr1a1a/akr1a1a/hkdc1/galm/gck/aldh9a1a.2/pfkmb | dre:560753/dre:560753/dre:100005587/dre:116517/dre:100536795/dre:568001/dre:406473/dre:368239/dre:561416/dre:406339/dre:561416/dre:393427/dre:406702/dre:445326/dre:445326/dre:321224/dre:436646/dre:751668/dre:100006238/dre:568001 | 20 | 10 | 560753\_1/116517/406473/368239/561416\_1/406702/445326\_1/321224/751668/568001\_3 | 10 | 560753/100005587\_1/100536795/568001\_2/406339\_1/561416/393427/445326/436646/100006238\_1 |
| dre00591 | Linoleic acid metabolism | 9/900 | 33/8379 | 0.0065218 | 0.1717395 | 406370\_1/406370/393592/553969/797309/560649/399485/767688/100148818 | pla2g6/pla2g6/cyp2p9/cyp3a65/cyp2j20/si:ch211-214p16.3/cyp2p10/zgc:154040/si:ch73-55i23.1 | dre:406370/dre:406370/dre:393592/dre:553969/dre:797309/dre:560649/dre:399485/dre:767688/dre:100148818 | 9 | 4 | 406370/393592/553969/560649 | 5 | 406370\_1/797309/399485/767688/100148818 |
| dre03018 | RNA degradation | 20/900 | 108/8379 | 0.0102807 | 0.1994695 | 373085\_1/448949\_1/568001\_2/394064\_1/553665/324574/448949/553665\_1/566017/100538016/492479\_1/565000/565659/561416\_1/317641\_1/566017\_1/565659\_1/561416/321035\_1/568001\_3 | hspa9/cnot1/pfkmb/exosc10/cnot6l/wu:fc33b09/cnot1/cnot6l/lsm8/si:dkey-79d12.5/btg3/exosc3/edc4/pfkpb/zcchc7/lsm8/edc4/pfkpb/pabpc4/pfkmb | dre:373085/dre:448949/dre:568001/dre:394064/dre:553665/dre:324574/dre:448949/dre:553665/dre:566017/dre:100538016/dre:492479/dre:324574/dre:565659/dre:561416/dre:317641/dre:566017/dre:565659/dre:561416/dre:321035/dre:568001 | 20 | 13 | 373085\_1/448949\_1/394064\_1/553665\_1/100538016/492479\_1/565000/561416\_1/317641\_1/566017\_1/565659\_1/321035\_1/568001\_3 | 7 | 568001\_2/553665/324574/448949/566017/565659/561416 |
| dre00380 | Tryptophan metabolism | 14/900 | 67/8379 | 0.0105492 | 0.1994695 | 100005587\_1/100536795/103911233/368262/447801\_1/393860\_1/447801/368239/571201/393860/571967/555401/103911241/100006238\_1 | aldh9a1a.1/wu:fb14d09/LOC103911233/dlst/aldh8a1/gcdha/aldh8a1/aldh2.2/si:ch73-97h19.2/gcdha/ido1/aoc1/LOC103911241/aldh9a1a.2 | dre:100005587/dre:100536795/dre:103911233/dre:368262/dre:447801/dre:393860/dre:447801/dre:368239/dre:571201/dre:393860/dre:571967/dre:555401/dre:571201/dre:100006238 | 14 | 7 | 103911233/447801\_1/368239/393860/571967/555401/103911241 | 7 | 100005587\_1/100536795/368262/393860\_1/447801/571201/100006238\_1 |
| dre01230 | Biosynthesis of amino acids | 20/900 | 109/8379 | 0.0113622 | 0.1994695 | 334195\_1/560753\_1/560753/568001\_2/322670\_1/550579/560827\_1/550524\_1/407673/100006589\_1/550579\_1/393799/561416\_1/445818/561416/406688/563642/334195/678634/568001\_3 | mat1a/tpi1b/tpi1b/pfkmb/aco2/idh3g/prps1b/cbsa/rpia/idh1/idh3g/pycr1a/pfkpb/cthl/pfkpb/got2a/sdsl/mat1a/mat2al/pfkmb | dre:334195/dre:560753/dre:560753/dre:568001/dre:322670/dre:550579/dre:560827/dre:550524/dre:407673/dre:100006589/dre:550579/dre:393799/dre:561416/dre:445818/dre:561416/dre:406688/dre:563642/dre:334195/dre:678634/dre:568001 | 20 | 10 | 334195\_1/560753\_1/560827\_1/100006589\_1/550579\_1/393799/561416\_1/445818/563642/568001\_3 | 10 | 560753/568001\_2/322670\_1/550579/550524\_1/407673/561416/406688/334195/678634 |
| dre00260 | Glycine, serine and threonine metabolism | 12/900 | 56/8379 | 0.0141678 | 0.2238506 | 394103\_1/541429\_1/541429/550524\_1/541329/541329\_1/445818/394103/405800/619259/563642/64607\_1 | sardh/aoc2/aoc2/cbsa/gcsha/gcsha/cthl/sardh/dao.2/dao.1/sdsl/alas2 | dre:394103/dre:541429/dre:541429/dre:550524/dre:541329/dre:541329/dre:445818/dre:394103/dre:405800/dre:619259/dre:563642/dre:64607 | 12 | 7 | 541429/541329/445818/394103/405800/619259/563642 | 5 | 394103\_1/541429\_1/550524\_1/541329\_1/64607\_1 |
| dre00900 | Terpenoid backbone biosynthesis | 7/900 | 26/8379 | 0.0170190 | 0.2293485 | 334380\_1/559054\_1/334380/559054/557720\_1/100003383\_1/100003383 | zmpste24/hmgcra/zmpste24/hmgcra/rce1a/pmvk/pmvk | dre:334380/dre:559054/dre:334380/dre:559054/dre:557720/dre:100003383/dre:100003383 | 7 | 4 | 334380\_1/559054\_1/557720\_1/100003383 | 3 | 334380/559054/100003383\_1 |
| dre04150 | mTOR signaling pathway | 38/900 | 251/8379 | 0.0178197 | 0.2293485 | 324140\_1/324140/796322/336619/436718\_1/100333504\_1/436718/79380/359840\_1/100000252\_1/573492/792689\_1/799099\_1/79380\_1/359840/100000252\_3/100006675/553214\_1/245702\_1/563708/556087\_1/555584/100330617\_1/58072/30369\_1/555584\_1/791993/790938/30127\_1/100000252/359839/245702/797250/100009657\_1/556087/58072\_1/497384\_1/573492\_1 | sgk1/sgk1/slc3a2a/eif4bb/rhocb/lamtor5/rhocb/eif4ea/atp6v1b2/pik3r3b/rragca/mlst8/lpin1/eif4ea/atp6v1b2/pik3r3b/prkcba/eif4ba/igf1rb/skp2/wnt2bb/prr5a/tsc1b/fzd8b/fzd7a/prr5a/wnt4b/zgc:158423/wnt2/pik3r3b/atp6v1ba/igf1rb/slc7a5/rps6ka3b/wnt2bb/fzd8b/prkca/rragca | dre:324140/dre:324140/dre:796322/dre:336619/dre:436718/dre:100333504/dre:436718/dre:79380/dre:359840/dre:100000252/dre:573492/dre:792689/dre:799099/dre:79380/dre:359840/dre:100000252/dre:100006675/dre:553214/dre:245702/dre:563708/dre:556087/dre:555584/dre:100330617/dre:58072/dre:30369/dre:555584/dre:791993/dre:790938/dre:30127/dre:100000252/dre:359839/dre:245702/dre:797250/dre:100009657/dre:556087/dre:58072/dre:497384/dre:573492 | 38 | 18 | 324140\_1/796322/436718/79380/799099\_1/359840/100000252\_3/100006675/553214\_1/100330617\_1/555584\_1/100000252/359839/245702/100009657\_1/556087/58072\_1/573492\_1 | 20 | 324140/336619/436718\_1/100333504\_1/359840\_1/100000252\_1/573492/792689\_1/79380\_1/245702\_1/563708/556087\_1/555584/58072/30369\_1/791993/790938/30127\_1/797250/497384\_1 |
| dre00030 | Pentose phosphate pathway | 10/900 | 45/8379 | 0.0188704 | 0.2293485 | 568001\_2/560827\_1/406762\_1/403070\_1/570579\_1/407673/403070/561416\_1/561416/568001\_3 | pfkmb/prps1b/pgd/rgn/g6pd/rpia/rgn/pfkpb/pfkpb/pfkmb | dre:568001/dre:560827/dre:406762/dre:403070/dre:570579/dre:407673/dre:403070/dre:561416/dre:561416/dre:568001 | 10 | 6 | 560827\_1/406762\_1/403070\_1/570579\_1/561416\_1/568001\_3 | 4 | 568001\_2/407673/403070/561416 |
| dre03440 | Homologous recombination | 10/900 | 46/8379 | 0.0218681 | 0.2464456 | 558923\_1/558923/406487/794038\_1/554171/404608/794038/406721/554171\_1/450081 | palb2/palb2/rad51/brip1/rad52/rad51d/brip1/rad51b/rad52/rad51c | dre:558923/dre:558923/dre:406487/dre:794038/dre:554171/dre:404608/dre:794038/dre:406721/dre:554171/dre:450081 | 10 | 7 | 558923/406487/404608/794038/406721/554171\_1/450081 | 3 | 558923\_1/794038\_1/554171 |
| dre00360 | Phenylalanine metabolism | 6/900 | 22/8379 | 0.0249565 | 0.2464456 | 541429\_1/541429/561410\_1/445287/394142/406688 | aoc2/aoc2/tat/hpdb/hpda/got2a | dre:541429/dre:541429/dre:561410/dre:445287/dre:394142/dre:406688 | 6 | 4 | 541429/561410\_1/445287/394142 | 2 | 541429\_1/406688 |
| dre00450 | Selenocompound metabolism | 6/900 | 22/8379 | 0.0249565 | 0.2464456 | 103911233/777719/571201/101884223/445818/103911241 | LOC103911233/papss2a/si:ch73-97h19.2/LOC101884223/cthl/LOC103911241 | dre:103911233/dre:777719/dre:571201/dre:101884223/dre:445818/dre:571201 | 6 | 4 | 103911233/101884223/445818/103911241 | 2 | 777719/571201 |
| dre00620 | Pyruvate metabolism | 13/900 | 68/8379 | 0.0271514 | 0.2523481 | 335715/100005587\_1/116517/100536795/445233/406473/368239/393427/559403\_1/406702/445326\_1/445326/100006238\_1 | mdh1ab/aldh9a1a.1/adh5/wu:fb14d09/me2/pck2/aldh2.2/zgc:63568/acaca/pdha1a/akr1a1a/akr1a1a/aldh9a1a.2 | dre:335715/dre:100005587/dre:116517/dre:100536795/dre:445233/dre:406473/dre:368239/dre:393427/dre:559403/dre:406702/dre:445326/dre:445326/dre:100006238 | 13 | 8 | 335715/116517/445233/406473/368239/559403\_1/406702/445326\_1 | 5 | 100005587\_1/100536795/393427/445326/100006238\_1 |
| dre00052 | Galactose metabolism | 9/900 | 43/8379 | 0.0361638 | 0.3105070 | 568001\_2/561416\_1/406339\_1/450083/561416/321224/436646/751668/568001\_3 | pfkmb/pfkpb/hk2/gla/pfkpb/hkdc1/galm/gck/pfkmb | dre:568001/dre:561416/dre:406339/dre:450083/dre:561416/dre:321224/dre:436646/dre:751668/dre:568001 | 9 | 5 | 561416\_1/450083/321224/751668/568001\_3 | 4 | 568001\_2/406339\_1/561416/436646 |
| dre00350 | Tyrosine metabolism | 10/900 | 50/8379 | 0.0373395 | 0.3105070 | 116517/541429\_1/541429/561410\_1/445287/394142/406688/393427/569363/30505 | adh5/aoc2/aoc2/tat/hpdb/hpda/got2a/zgc:63568/tpo/dbh | dre:116517/dre:541429/dre:541429/dre:561410/dre:445287/dre:394142/dre:406688/dre:393427/dre:569363/dre:30505 | 10 | 6 | 116517/541429/561410\_1/445287/394142/30505 | 4 | 541429\_1/406688/393427/569363 |
| dre00500 | Starch and sucrose metabolism | 9/900 | 44/8379 | 0.0413011 | 0.3262783 | 393444/406539/445049/100333665/406339\_1/100333665\_1/321224/561803/751668 | pygmb/amy2a/zgc:92137/gbe1a/hk2/gbe1a/hkdc1/gyg2/gck | dre:393444/dre:406539/dre:445049/dre:100333665/dre:406339/dre:100333665/dre:321224/dre:561803/dre:751668 | 9 | 6 | 393444/406539/445049/100333665/321224/751668 | 3 | 406339\_1/100333665\_1/561803 |
| dre00053 | Ascorbate and aldarate metabolism | 12/900 | 66/8379 | 0.0464965 | 0.3384483 | 100005587\_1/100536795/403070\_1/368239/403070/406731/100415796/570913\_1/445326\_1/445326/767756/100006238\_1 | aldh9a1a.1/wu:fb14d09/rgn/aldh2.2/rgn/ugt1ab/ugt5b5/ugt5g1/akr1a1a/akr1a1a/ugt5a2/aldh9a1a.2 | dre:100005587/dre:100536795/dre:403070/dre:368239/dre:403070/dre:406731/dre:678597/dre:570913/dre:445326/dre:445326/dre:767756/dre:100006238 | 12 | 3 | 403070\_1/368239/445326\_1 | 9 | 100005587\_1/100536795/403070/406731/100415796/570913\_1/445326/767756/100006238\_1 |
| dre04070 | Phosphatidylinositol signaling system | 24/900 | 157/8379 | 0.0471257 | 0.3384483 | 724010/100002015\_1/336121/724010\_1/368717/368717\_1/100000252\_1/337236\_1/337236\_2/559938\_1/100000252\_3/100006675/337236/566642\_1/566642/559938/571356/678558\_1/571356\_1/571356\_2/559198\_1/100000252/559198/497384\_1 | dgkaa/cds1/calm2a/dgkaa/itpka/itpka/pik3r3b/synj1/synj1/ppip5k1b/pik3r3b/prkcba/synj1/tmem55a/tmem55a/ppip5k1b/pik3c2a/mtmr7a/pik3c2a/pik3c2a/mtmr7b/pik3r3b/mtmr7b/prkca | dre:724010/dre:100002015/dre:192322/dre:724010/dre:368717/dre:368717/dre:100000252/dre:337236/dre:337236/dre:559938/dre:100000252/dre:100006675/dre:337236/dre:566642/dre:566642/dre:559938/dre:571356/dre:678558/dre:571356/dre:571356/dre:559198/dre:100000252/dre:559198/dre:497384 | 24 | 15 | 100002015\_1/336121/724010\_1/368717/337236\_2/559938\_1/100000252\_3/100006675/337236/566642\_1/571356/678558\_1/571356\_2/100000252/559198 | 9 | 724010/368717\_1/100000252\_1/337236\_1/566642/559938/571356\_1/559198\_1/497384\_1 |
| dre00592 | alpha-Linolenic acid metabolism | 6/900 | 26/8379 | 0.0533315 | 0.3663645 | 406370\_1/406370/140615/560649/767688/100148818 | pla2g6/pla2g6/fads2/si:ch211-214p16.3/zgc:154040/si:ch73-55i23.1 | dre:406370/dre:406370/dre:140615/dre:560649/dre:767688/dre:100148818 | 6 | 3 | 406370/140615/560649 | 3 | 406370\_1/767688/100148818 |
| dre00051 | Fructose and mannose metabolism | 10/900 | 54/8379 | 0.0590947 | 0.3765745 | 560753\_1/560753/568001\_2/561416\_1/406339\_1/436909/561416/100007686/321224/568001\_3 | tpi1b/tpi1b/pfkmb/pfkpb/hk2/khk/pfkpb/LOC100007686/hkdc1/pfkmb | dre:560753/dre:560753/dre:568001/dre:561416/dre:406339/dre:436909/dre:561416/dre:100007686/dre:321224/dre:568001 | 10 | 5 | 560753\_1/561416\_1/436909/321224/568001\_3 | 5 | 560753/568001\_2/406339\_1/561416/100007686 |
| dre00020 | Citrate cycle (TCA cycle) | 9/900 | 47/8379 | 0.0595846 | 0.3765745 | 335715/322670\_1/550579/406473/368262/100006589\_1/550579\_1/317746/406702 | mdh1ab/aco2/idh3g/pck2/dlst/idh1/idh3g/suclg2/pdha1a | dre:335715/dre:322670/dre:550579/dre:406473/dre:368262/dre:100006589/dre:550579/dre:317746/dre:406702 | 9 | 6 | 335715/406473/100006589\_1/550579\_1/317746/406702 | 3 | 322670\_1/550579/368262 |
| dre03460 | Fanconi anemia pathway | 11/900 | 63/8379 | 0.0703734 | 0.4245372 | 558923\_1/100538153/558923/406487/794038\_1/402936/100538153\_1/794038/402936\_1/450081/570043\_1 | palb2/slx4/palb2/rad51/brip1/fancg/slx4/brip1/fancg/rad51c/rev1 | dre:558923/dre:100538153/dre:558923/dre:406487/dre:794038/dre:402936/dre:100538153/dre:794038/dre:402936/dre:450081/dre:570043 | 11 | 7 | 100538153/558923/406487/794038/402936\_1/450081/570043\_1 | 4 | 558923\_1/794038\_1/402936/100538153\_1 |
| dre04130 | SNARE interactions in vesicular transport | 10/900 | 56/8379 | 0.0725475 | 0.4245372 | 567385\_1/449923/436605/449923\_1/393836/571872/393294/325484/393236/565822\_1 | vti1a/si:ch73-234b20.5/stx5a/si:ch73-234b20.5/stx11b.1/zgc:165520/use1/vamp5/sybl1/bnip1a | dre:567385/dre:449923/dre:436605/dre:449923/dre:393836/dre:571872/dre:393294/dre:325484/dre:393236/dre:565822 | 10 | 9 | 567385\_1/449923/436605/449923\_1/393836/571872/325484/393236/565822\_1 | 1 | 393294 |
| dre04810 | Regulation of actin cytoskeleton | 52/900 | 404/8379 | 0.0931454 | 0.5213732 | 57934\_1/57934/286739\_1/799354/30429\_2/793158/570868\_1/436718\_1/286739/566764/436718/566764\_1/100000252\_1/557935/793158\_1/386856\_1/100000252\_3/799354\_1/100331581/100002872\_2/100005049/novel.946/677750/100331581\_1/569566/571431/560564\_2/556226\_1/796490\_2/100536705\_2/494456/100005469\_1/553493/570832/323285\_1/378480/553493\_1/393611/445305\_1/30429/58075/100000252/767646\_1/794365/103909601/797491\_1/564565/798694\_1/570832\_1/564266/792699/101882911 | actb1/actb1/msna/cxcl12b/mylpfa/cdc42l/itga1/rhocb/msna/scin/rhocb/scin/pik3r3b/zmp:0000001082/cdc42l/pdgfra/pik3r3b/cxcl12b/si:dkey-44g17.6/cyfip2/fgf1b/-/myl2b/si:dkey-44g17.6/LOC569566/LOC571431/pdgfc/cdc42l2/pdgfbb/itgae.2/fgf23/fn1a/arhgef7a/c7b/scinla/pdgfaa/arhgef7a/c6/arpc4l/mylpfa/fgd/pik3r3b/abi2a/si:dkey-163m14.7/LOC103909601/itgb7/arhgap35/LOC798694/c7b/itgb3a/rac1l/LOC101882911 | dre:57934/dre:57934/dre:286739/dre:799354/dre:30429/dre:793158/dre:570868/dre:436718/dre:286739/dre:566764/dre:436718/dre:566764/dre:100000252/dre:557935/dre:793158/dre:386856/dre:100000252/dre:799354/dre:100331581/dre:100002872/dre:100005049/dre:386856/dre:677750/dre:100331581/dre:569566/dre:571431/dre:560564/dre:556226/dre:796490/dre:100536705/dre:494456/dre:100005469/dre:553493/dre:570832/dre:323285/dre:103909601/dre:553493/dre:393611/dre:445305/dre:30429/dre:58075/dre:100000252/dre:767646/dre:794365/dre:103909601/dre:797491/dre:564565/dre:798694/dre:570832/dre:564266/dre:792699/dre:321070 | 52 | 27 | 57934\_1/799354/570868\_1/286739/436718/566764\_1/793158\_1/100000252\_3/100331581/100002872\_2/677750/556226\_1/796490\_2/494456/100005469\_1/570832/553493\_1/30429/58075/100000252/794365/103909601/564565/798694\_1/570832\_1/564266/101882911 | 25 | 57934/286739\_1/30429\_2/793158/436718\_1/566764/100000252\_1/557935/386856\_1/799354\_1/100005049/novel.946/100331581\_1/569566/571431/560564\_2/100536705\_2/553493/323285\_1/378480/393611/445305\_1/767646\_1/797491\_1/792699 |
| dre03430 | Mismatch repair | 6/900 | 30/8379 | 0.0956951 | 0.5213732 | 556995\_1/568323/556995\_2/565400/568323\_1/565400\_1 | lig1/mlh3/lig1/msh3/mlh3/msh3 | dre:556995/dre:568323/dre:556995/dre:565400/dre:568323/dre:565400 | 6 | 4 | 556995\_1/565400/568323\_1/565400\_1 | 2 | 568323/556995\_2 |
| dre01232 | Nucleotide metabolism | 19/900 | 131/8379 | 0.1068928 | 0.5480363 | 100330864/492517\_1/334431\_1/492517/100005763\_1/550560\_1/100005763/437016\_1/103910413\_1/393744\_1/553639\_1/103910413/402953\_1/333997/558466\_1/100005067\_1/393744\_2/447905/333997\_1 | LOC100330864/zgc:103586/adsl/zgc:103586/uck2a/entpd6/uck2a/tk2/tymp/dctpp1/gmpr/tymp/pnp6/ampd3b/uckl1b/dguok/dctpp1/entpd2a.1/ampd3b | dre:100330864/dre:492517/dre:334431/dre:492517/dre:100005763/dre:550560/dre:100005763/dre:437016/dre:103910413/dre:393744/dre:553639/dre:103910413/dre:402953/dre:333997/dre:558466/dre:100005067/dre:393744/dre:447905/dre:333997 | 19 | 13 | 100330864/334431\_1/492517/550560\_1/100005763/103910413\_1/393744\_1/553639\_1/402953\_1/333997/558466\_1/447905/333997\_1 | 6 | 492517\_1/100005763\_1/437016\_1/103910413/100005067\_1/393744\_2 |
| dre00100 | Steroid biosynthesis | 5/900 | 24/8379 | 0.1075261 | 0.5480363 | 322481/100004700/565117/406662\_2/494054\_1 | cel.1/cyp24a1/cel.2/msmo1/tm7sf2 | dre:322481/dre:100004700/dre:565117/dre:406662/dre:494054 | 5 | 4 | 322481/100004700/565117/406662\_2 | 1 | 494054\_1 |
| dre04210 | Apoptosis | 31/900 | 232/8379 | 0.1169196 | 0.5772906 | 57934\_1/57934/140621/373107\_1/431763\_1/791531\_1/431763/100000252\_1/556410\_1/100000252\_3/552927\_1/394198/570238/567021/569298/564295\_2/140621\_1/101886621\_1/768248/58100\_1/561021/559564/552927/58133/431777/791531/100000252/561021\_1/100135062\_2/246726/558237\_1 | actb1/actb1/casp3a/birc2/gadd45ab/casp6l1/gadd45ab/pik3r3b/atf4b/pik3r3b/casp6/fosab/fosaa/ern2/ctsbb/traf2a/casp3a/LOC101886621/fas/badb/si:dkey-103e21.5/eif2ak3/casp6/ngfb/tuba7l/casp6l1/pik3r3b/si:dkey-103e21.5/csf2rb/birc5b/dab2ipb | dre:57934/dre:57934/dre:140621/dre:373107/dre:431763/dre:791531/dre:431763/dre:100000252/dre:556410/dre:100000252/dre:552927/dre:394198/dre:570238/dre:567021/dre:569298/dre:564295/dre:140621/dre:101886621/dre:768248/dre:58100/dre:561021/dre:559564/dre:552927/dre:58133/dre:431777/dre:791531/dre:100000252/dre:561021/dre:100135062/dre:246726/dre:558237 | 31 | 17 | 57934\_1/431763/556410\_1/100000252\_3/394198/570238/567021/569298/101886621\_1/768248/561021/552927/58133/431777/791531/100000252/100135062\_2 | 14 | 57934/140621/373107\_1/431763\_1/791531\_1/100000252\_1/552927\_1/564295\_2/140621\_1/58100\_1/559564/561021\_1/246726/558237\_1 |
| dre03015 | mRNA surveillance pathway | 17/900 | 118/8379 | 0.1277218 | 0.5864549 | 386630/324267/386630\_1/324267\_1/393693\_1/100005893/393693/100005893\_1/492341\_1/394058\_1/563172/100002680\_2/562282\_1/321035\_1/559392\_1/562282/559392 | tardbpl/hbs1l/tardbpl/hbs1l/sap18/magoh/sap18/magoh/pcf11/fus/etf1a/msi2a/ppp2r5b/pabpc4/ppp2r2cb/ppp2r5b/ppp2r2cb | dre:386630/dre:324267/dre:386630/dre:324267/dre:393693/dre:100005893/dre:393693/dre:100005893/dre:492341/dre:394058/dre:563172/dre:100002680/dre:562282/dre:321035/dre:559392/dre:562282/dre:559392 | 17 | 8 | 386630\_1/324267\_1/393693\_1/100005893/100002680\_2/321035\_1/559392\_1/562282 | 9 | 386630/324267/393693/100005893\_1/492341\_1/394058\_1/563172/562282\_1/559392 |
| dre00470 | D-Amino acid metabolism | 3/900 | 12/8379 | 0.1303694 | 0.5864549 | 560387/405800/619259 | c17h14orf159/dao.2/dao.1 | dre:560387/dre:405800/dre:619259 | 3 | 3 | 560387/405800/619259 | 0 |  |
| dre00920 | Sulfur metabolism | 3/900 | 12/8379 | 0.1303694 | 0.5864549 | 777719/393542\_1/566675 | papss2a/selenbp1/suox | dre:777719/dre:393542/dre:566675 | 3 | 1 | 393542\_1 | 2 | 777719/566675 |
| dre02010 | ABC transporters | 10/900 | 63/8379 | 0.1336226 | 0.5864549 | 368620\_1/406803/553281/570453\_1/406803\_1/406803\_2/571189/570148\_1/735310/556826\_1 | abcc4/abcd3a/abcc8/abcg2b/abcd3a/abcd3a/abcb11b/abcb9/abcg2d/si:dkey-57h18.2 | dre:368620/dre:406803/dre:553281/dre:570453/dre:406803/dre:406803/dre:571189/dre:570148/dre:735310/dre:556826 | 10 | 8 | 368620\_1/570453\_1/406803\_1/406803\_2/571189/570148\_1/735310/556826\_1 | 2 | 406803/553281 |
| dre04370 | VEGF signaling pathway | 15/900 | 104/8379 | 0.1448226 | 0.6184316 | 793158/565241\_2/559020/562552/100000252\_1/793158\_1/100000252\_3/100006675/556226\_1/58100\_1/565241/100148818/100000252/497384\_1/792699 | cdc42l/ppp3ccb/ptgs2b/zgc:171775/pik3r3b/cdc42l/pik3r3b/prkcba/cdc42l2/badb/ppp3ccb/si:ch73-55i23.1/pik3r3b/prkca/rac1l | dre:793158/dre:565241/dre:559020/dre:562552/dre:100000252/dre:793158/dre:100000252/dre:100006675/dre:556226/dre:58100/dre:565241/dre:100148818/dre:100000252/dre:497384/dre:792699 | 15 | 8 | 559020/562552/793158\_1/100000252\_3/100006675/556226\_1/565241/100000252 | 7 | 793158/565241\_2/100000252\_1/58100\_1/100148818/497384\_1/792699 |
| dre00561 | Glycerolipid metabolism | 13/900 | 89/8379 | 0.1553178 | 0.6311882 | 724010/100005587\_1/100536795/724010\_1/322481/368239/799099\_1/565117/450083/445326\_1/572841/445326/100006238\_1 | dgkaa/aldh9a1a.1/wu:fb14d09/dgkaa/cel.1/aldh2.2/lpin1/cel.2/gla/akr1a1a/mboat1/akr1a1a/aldh9a1a.2 | dre:724010/dre:100005587/dre:100536795/dre:724010/dre:322481/dre:368239/dre:799099/dre:565117/dre:450083/dre:445326/dre:572841/dre:445326/dre:100006238 | 13 | 8 | 724010\_1/322481/368239/799099\_1/565117/450083/445326\_1/572841 | 5 | 724010/100005587\_1/100536795/445326/100006238\_1 |
| dre00330 | Arginine and proline metabolism | 12/900 | 81/8379 | 0.1557996 | 0.6311882 | 100005587\_1/100536795/321892/321892\_1/368239/393799/405800/406688/555401/619259/327506\_1/100006238\_1 | aldh9a1a.1/wu:fb14d09/ckmt1/ckmt1/aldh2.2/pycr1a/dao.2/got2a/aoc1/dao.1/ckmt2a/aldh9a1a.2 | dre:100005587/dre:100536795/dre:321892/dre:321892/dre:368239/dre:393799/dre:405800/dre:406688/dre:555401/dre:619259/dre:327506/dre:100006238 | 12 | 7 | 321892\_1/368239/393799/405800/555401/619259/327506\_1 | 5 | 100005587\_1/100536795/321892/406688/100006238\_1 |
| dre00603 | Glycosphingolipid biosynthesis - globo and isoglobo series | 4/900 | 20/8379 | 0.1605074 | 0.6340043 | 447881/450083/100093713/100005375 | gbgt1l3/gla/zgc:165519/si:ch211-135f11.4 | dre:447881/dre:450083/dre:100093713/dre:100005375 | 4 | 3 | 447881/450083/100005375 | 1 | 100093713 |
| dre03410 | Base excision repair | 9/900 | 58/8379 | 0.1651686 | 0.6365035 | 321622/110438871/393774\_2/393774/556995\_1/556995\_2/553405\_1/558198\_1/796446\_1 | hmgb1a/LOC110438871/pole3/pole3/lig1/lig1/pole/si:ch211-141o9.10/adprhl2 | dre:110438871/dre:110438871/dre:393774/dre:393774/dre:556995/dre:556995/dre:553405/dre:558198/dre:796446 | 9 | 3 | 110438871/393774/556995\_1 | 6 | 321622/393774\_2/556995\_2/553405\_1/558198\_1/796446\_1 |
| dre00512 | Mucin type O-glycan biosynthesis | 7/900 | 43/8379 | 0.1731063 | 0.6417131 | 324510\_1/324510/557675/557675\_1/555344/337131/568500 | gcnt4a/gcnt4a/c1galt1a/c1galt1a/si:dkey-202e17.1/c1galt1b/galnt8a.2 | dre:324510/dre:324510/dre:557675/dre:557675/dre:555344/dre:337131/dre:568500 | 7 | 4 | 324510\_1/557675\_1/555344/337131 | 3 | 324510/557675/568500 |
| dre00983 | Drug metabolism - other enzymes | 17/900 | 124/8379 | 0.1746434 | 0.6417131 | 100330864/492517\_1/492517/406731/100005763\_1/100005763/437016\_1/794381/103910413\_1/100415796/393493/570913\_1/406703/103910413/558466\_1/431762\_1/767756 | LOC100330864/zgc:103586/zgc:103586/ugt1ab/uck2a/uck2a/tk2/si:dkey-78a14.4/tymp/ugt5b5/gstt2/ugt5g1/gsta.1/tymp/uckl1b/mgst1.2/ugt5a2 | dre:100330864/dre:492517/dre:492517/dre:406731/dre:100005763/dre:100005763/dre:437016/dre:794381/dre:103910413/dre:678597/dre:393493/dre:570913/dre:406703/dre:103910413/dre:558466/dre:431762/dre:767756 | 17 | 7 | 100330864/492517/100005763/103910413\_1/393493/558466\_1/431762\_1 | 10 | 492517\_1/406731/100005763\_1/437016\_1/794381/100415796/570913\_1/406703/103910413/767756 |
| dre00130 | Ubiquinone and other terpenoid-quinone biosynthesis | 3/900 | 14/8379 | 0.1843234 | 0.6560992 | 561410\_1/445287/394142 | tat/hpdb/hpda | dre:561410/dre:445287/dre:394142 | 3 | 3 | 561410\_1/445287/394142 | 0 |  |
| dre00240 | Pyrimidine metabolism | 12/900 | 84/8379 | 0.1868637 | 0.6560992 | 100330864/492517\_1/492517/100005763\_1/550560\_1/100005763/437016\_1/103910413\_1/393744\_1/103910413/558466\_1/393744\_2 | LOC100330864/zgc:103586/zgc:103586/uck2a/entpd6/uck2a/tk2/tymp/dctpp1/tymp/uckl1b/dctpp1 | dre:100330864/dre:492517/dre:492517/dre:100005763/dre:550560/dre:100005763/dre:437016/dre:103910413/dre:393744/dre:103910413/dre:558466/dre:393744 | 12 | 7 | 100330864/492517/550560\_1/100005763/103910413\_1/393744\_1/558466\_1 | 5 | 492517\_1/100005763\_1/437016\_1/103910413/393744\_2 |
| dre00310 | Lysine degradation | 14/900 | 102/8379 | 0.2024193 | 0.6923893 | 100005587\_1/100536795/368262/393860\_1/368239/393860/556077\_1/556535\_1/100003615/100003615\_1/572649/402830/100006238\_1/556535 | aldh9a1a.1/wu:fb14d09/dlst/gcdha/aldh2.2/gcdha/plod3/setd1a/prdm2a/prdm2a/bbox1/ehmt1b/aldh9a1a.2/setd1a | dre:100005587/dre:100536795/dre:368262/dre:393860/dre:368239/dre:393860/dre:556077/dre:556535/dre:100003615/dre:100003615/dre:572649/dre:402830/dre:100006238/dre:556535 | 14 | 7 | 368239/393860/556077\_1/100003615/572649/402830/556535 | 7 | 100005587\_1/100536795/368262/393860\_1/556535\_1/100003615\_1/100006238\_1 |
| dre05132 | Salmonella infection | 49/900 | 406/8379 | 0.2087747 | 0.6923893 | 57934\_1/386590\_1/386590/57934/562529\_1/30429\_2/793158/140621/436718\_1/373107\_1/100004870\_1/562372\_1/558917\_1/562552/436718/563023\_1/793158\_1/406811\_1/394198/570238/798985/563023/436734\_1/798985\_1/100002872\_2/101882034/677750/566185/562372/393653\_1/564295\_2/140621\_1/556226\_1/571356/571356\_1/561021/571356\_2/novel.848/110437957/548603\_1/431777/100002946/445305\_1/30429/561021\_1/403131/792699/799806\_2/101882911 | actb1/hsp90b1/hsp90b1/actb1/flna/mylpfa/cdc42l/casp3a/rhocb/birc2/exoc2/snx18a/snx9b/zgc:171775/rhocb/flnb/cdc42l/zgc:55461/fosab/fosaa/vps16/flnb/txn/vps16/cyfip2/LOC101882034/myl2b/caspbl/snx18a/rhogd/traf2a/casp3a/cdc42l2/pik3c2a/pik3c2a/si:dkey-103e21.5/pik3c2a/-/LOC110437957/snx18b/tuba7l/cxcl8a/arpc4l/mylpfa/si:dkey-103e21.5/tlr4ba/rac1l/anxa2b/LOC101882911 | dre:57934/dre:386590/dre:386590/dre:57934/dre:562529/dre:30429/dre:793158/dre:140621/dre:436718/dre:373107/dre:100004870/dre:562372/dre:558917/dre:562552/dre:436718/dre:563023/dre:793158/dre:336681/dre:394198/dre:570238/dre:798985/dre:563023/dre:436734/dre:798985/dre:100002872/dre:406297/dre:677750/dre:566185/dre:562372/dre:393653/dre:564295/dre:140621/dre:556226/dre:571356/dre:571356/dre:561021/dre:571356/dre:393993/dre:373874/dre:548603/dre:431777/dre:100002946/dre:445305/dre:30429/dre:561021/dre:403131/dre:792699/dre:799806/dre:321070 | 49 | 27 | 57934\_1/386590\_1/562529\_1/100004870\_1/562372\_1/558917\_1/562552/436718/563023\_1/793158\_1/394198/570238/798985\_1/100002872\_2/101882034/677750/566185/393653\_1/556226\_1/571356/561021/571356\_2/110437957/548603\_1/431777/30429/101882911 | 22 | 386590/57934/30429\_2/793158/140621/436718\_1/373107\_1/406811\_1/798985/563023/436734\_1/562372/564295\_2/140621\_1/571356\_1/novel.848/100002946/445305\_1/561021\_1/403131/792699/799806\_2 |
| dre00340 | Histidine metabolism | 5/900 | 30/8379 | 0.2147283 | 0.6923893 | 100005587\_1/100536795/368239/555401/100006238\_1 | aldh9a1a.1/wu:fb14d09/aldh2.2/aoc1/aldh9a1a.2 | dre:100005587/dre:100536795/dre:368239/dre:555401/dre:100006238 | 5 | 2 | 368239/555401 | 3 | 100005587\_1/100536795/100006238\_1 |
| dre01210 | 2-Oxocarboxylic acid metabolism | 5/900 | 30/8379 | 0.2147283 | 0.6923893 | 322670\_1/550579/100006589\_1/550579\_1/406688 | aco2/idh3g/idh1/idh3g/got2a | dre:322670/dre:550579/dre:100006589/dre:550579/dre:406688 | 5 | 2 | 100006589\_1/550579\_1 | 3 | 322670\_1/550579/406688 |
| dre04120 | Ubiquitin mediated proteolysis | 25/900 | 199/8379 | 0.2302529 | 0.7175345 | 550134/192341/564149/550134\_1/373107\_1/692325/692325\_1/564149\_1/novel.905/323951/323883/327215\_2/100136859/323951\_2/100001302/447935\_1/556277/563708/564866\_1/559439\_1/393223\_1/393445\_1/30100/559981/100151173\_1 | ubb/tceb2/pias2/ubb/birc2/flj11011l/flj11011l/pias2/-/klhl13/cul1a/cul5a/fbxw8/klhl13/uba7/tceb1a/cblb/skp2/trip12/huwe1/fzr1a/ube3c/ube2kb/si:ch73-190m4.1/si:rp71-68n21.9 | dre:550134/dre:192341/dre:564149/dre:550134/dre:373107/dre:692325/dre:692325/dre:564149/dre:777766/dre:323951/dre:323883/dre:327215/dre:100136859/dre:323951/dre:100001302/dre:447935/dre:556277/dre:563708/dre:564866/dre:559439/dre:393223/dre:393445/dre:30100/dre:559981/dre:100151173 | 25 | 12 | 192341/550134\_1/692325/564149\_1/novel.905/323951\_2/556277/564866\_1/559439\_1/393445\_1/559981/100151173\_1 | 13 | 550134/564149/373107\_1/692325\_1/323951/323883/327215\_2/100136859/100001302/447935\_1/563708/393223\_1/30100 |
| dre01240 | Biosynthesis of cofactors | 29/900 | 234/8379 | 0.2316092 | 0.7175345 | 334195\_1/100536795/334431\_1/557552/403070\_1/368239/403070/445287/406731/394142/571967/100004977\_2/445083/393580/100415796/100007686/570913\_1/406789/407663/445326\_1/64607\_1/110438097/100330410/574009/450084\_1/334195/445326/767756/678634 | mat1a/wu:fb14d09/adsl/zgc:153031/rgn/aldh2.2/rgn/hpdb/ugt1ab/hpda/ido1/mthfd2/dhrs3a/bco1l/ugt5b5/LOC100007686/ugt5g1/pank1a/si:ch211-107o10.3/akr1a1a/alas2/LOC110438097/alp3/alpi.2/gss/mat1a/akr1a1a/ugt5a2/mat2al | dre:334195/dre:100536795/dre:334431/dre:557552/dre:403070/dre:368239/dre:403070/dre:445287/dre:406731/dre:394142/dre:571967/dre:100004977/dre:445083/dre:393580/dre:678597/dre:100007686/dre:570913/dre:406789/dre:407663/dre:445326/dre:64607/dre:572070/dre:100330410/dre:574009/dre:450084/dre:334195/dre:445326/dre:767756/dre:678634 | 29 | 16 | 334195\_1/334431\_1/557552/403070\_1/368239/445287/394142/571967/445083/393580/406789/445326\_1/110438097/100330410/574009/450084\_1 | 13 | 100536795/403070/406731/100004977\_2/100415796/100007686/570913\_1/407663/64607\_1/334195/445326/767756/678634 |
| dre03030 | DNA replication | 7/900 | 48/8379 | 0.2529037 | 0.7640096 | 393241\_1/393774\_2/393774/556995\_1/393241\_2/556995\_2/553405\_1 | rnaseh2b/pole3/pole3/lig1/rnaseh2b/lig1/pole | dre:393241/dre:393774/dre:393774/dre:556995/dre:393241/dre:556995/dre:553405 | 7 | 3 | 393241\_1/393774/556995\_1 | 4 | 393774\_2/393241\_2/556995\_2/553405\_1 |
| dre05168 | Herpes simplex virus 1 infection | 27/900 | 220/8379 | 0.2579975 | 0.7640096 | 140621/373107\_1/100149189/100000252\_1/565338\_1/100000252\_3/30400/564295\_2/30762/140621\_1/406288\_1/58113/368878/100330617\_1/768248/58100\_1/561021/30763/559564/368481/30163/791704/100000252/571282/561021\_1/564266/556826\_1 | casp3a/birc2/si:ch73-237c6.1/pik3r3b/eif2ak1/pik3r3b/b2m/traf2a/mhc2dab/casp3a/srsf1a/cd74a/si:busm1-266f07.2/tsc1b/fas/badb/si:dkey-103e21.5/mhc2dcb/eif2ak3/stat1b/tapbp.1/zgc:153759/pik3r3b/LOC571282/si:dkey-103e21.5/itgb3a/si:dkey-57h18.2 | dre:140621/dre:373107/dre:100149189/dre:100000252/dre:565338/dre:100000252/dre:30400/dre:564295/dre:30762/dre:140621/dre:406288/dre:58113/dre:368878/dre:100330617/dre:768248/dre:58100/dre:561021/dre:30763/dre:559564/dre:368481/dre:30163/dre:791704/dre:100000252/dre:571282/dre:561021/dre:564266/dre:556826 | 27 | 14 | 100149189/100000252\_3/30762/58113/368878/100330617\_1/768248/561021/30763/791704/100000252/571282/564266/556826\_1 | 13 | 140621/373107\_1/100000252\_1/565338\_1/30400/564295\_2/140621\_1/406288\_1/58100\_1/559564/368481/30163/561021\_1 |
| dre04137 | Mitophagy - animal | 15/900 | 116/8379 | 0.2611172 | 0.7640096 | 550134/100000242/550134\_1/novel.905/556410\_1/555713\_1/555713/326974\_1/564996/492676/336159\_1/559564/561933\_2/561933/561933\_1 | ubb/zgc:92606/ubb/-/atf4b/nbr1b/nbr1b/gabarapa/tfe3b/tbc1d15/optn/eif2ak3/rhot1b/rhot1b/rhot1b | dre:550134/dre:100000242/dre:550134/dre:777766/dre:556410/dre:555713/dre:555713/dre:326974/dre:564996/dre:492676/dre:336159/dre:559564/dre:561933/dre:561933/dre:561933 | 15 | 9 | 550134\_1/novel.905/556410\_1/555713/326974\_1/564996/492676/561933\_2/561933\_1 | 6 | 550134/100000242/555713\_1/336159\_1/559564/561933 |
| dre00410 | beta-Alanine metabolism | 6/900 | 41/8379 | 0.2743520 | 0.7838325 | 100005587\_1/541429\_1/100536795/541429/368239/100006238\_1 | aldh9a1a.1/aoc2/wu:fb14d09/aoc2/aldh2.2/aldh9a1a.2 | dre:100005587/dre:541429/dre:100536795/dre:541429/dre:368239/dre:100006238 | 6 | 2 | 541429/368239 | 4 | 100005587\_1/541429\_1/100536795/100006238\_1 |
| dre03060 | Protein export | 4/900 | 25/8379 | 0.2782048 | 0.7838325 | 445299/436794\_2/436794/445299\_1 | immp2l/sec11a/sec11a/immp2l | dre:445299/dre:436794/dre:436794/dre:445299 | 4 | 2 | 436794/445299\_1 | 2 | 445299/436794\_2 |
| dre04115 | p53 signaling pathway | 13/900 | 101/8379 | 0.2866208 | 0.7838325 | 100330864/140621/431763\_1/431763/171473/436699/436699\_1/140621\_1/768248/561021/796473\_1/100320152/561021\_1 | LOC100330864/casp3a/gadd45ab/gadd45ab/ccng1/zgc:92658/zgc:92658/casp3a/fas/si:dkey-103e21.5/tp53i3/steap3/si:dkey-103e21.5 | dre:100330864/dre:140621/dre:431763/dre:431763/dre:171473/dre:436699/dre:436699/dre:140621/dre:768248/dre:561021/dre:796473/dre:100320152/dre:561021 | 13 | 6 | 100330864/431763/171473/436699\_1/768248/561021 | 7 | 140621/431763\_1/436699/140621\_1/796473\_1/100320152/561021\_1 |
| dre00565 | Ether lipid metabolism | 9/900 | 67/8379 | 0.2899305 | 0.7838325 | 406370\_1/406370/492735/565743\_1/560225/560649/767688/100148818/567170\_1 | pla2g6/pla2g6/si:ch211-212o1.2/pld2/cept1a/si:ch211-214p16.3/zgc:154040/si:ch73-55i23.1/pafah1b2 | dre:406370/dre:406370/dre:492735/dre:565743/dre:560225/dre:560649/dre:767688/dre:100148818/dre:567170 | 9 | 6 | 406370/492735/565743\_1/560225/560649/567170\_1 | 3 | 406370\_1/767688/100148818 |
| dre00400 | Phenylalanine, tyrosine and tryptophan biosynthesis | 2/900 | 10/8379 | 0.2926970 | 0.7838325 | 561410\_1/406688 | tat/got2a | dre:561410/dre:406688 | 2 | 1 | 561410\_1 | 1 | 406688 |
| dre00740 | Riboflavin metabolism | 2/900 | 11/8379 | 0.3342154 | 0.8801004 | 436725\_1/436725 | acp5b/acp5b | dre:436725/dre:436725 | 2 | 1 | 436725\_1 | 1 | 436725 |
| dre00562 | Inositol phosphate metabolism | 15/900 | 123/8379 | 0.3410198 | 0.8832973 | 560753\_1/560753/368717/368717\_1/337236\_1/337236\_2/337236/100006223/571356/678558\_1/566479\_1/571356\_1/571356\_2/559198\_1/559198 | tpi1b/tpi1b/itpka/itpka/synj1/synj1/synj1/si:dkey-266f7.9/pik3c2a/mtmr7a/plch1/pik3c2a/pik3c2a/mtmr7b/mtmr7b | dre:560753/dre:560753/dre:368717/dre:368717/dre:337236/dre:337236/dre:337236/dre:100006223/dre:571356/dre:678558/dre:566479/dre:571356/dre:571356/dre:559198/dre:559198 | 15 | 9 | 560753\_1/368717/337236\_2/337236/100006223/571356/678558\_1/571356\_2/559198 | 6 | 560753/368717\_1/337236\_1/566479\_1/571356\_1/559198\_1 |
| dre03320 | PPAR signaling pathway | 13/900 | 106/8379 | 0.3494294 | 0.8884887 | 550134/550134\_1/406473/30355/723999/novel.905/334529/30355\_1/553512/140615/794841\_1/795106/550432 | ubb/ubb/pck2/apoa1a/zgc:136333/-/aqp7/apoa1a/slc27a1b/fads2/plin2/cyp27a1.2/slc27a4 | dre:550134/dre:550134/dre:406473/dre:30355/dre:795106/dre:777766/dre:334529/dre:30355/dre:553512/dre:140615/dre:794841/dre:795106/dre:550432 | 13 | 8 | 550134\_1/406473/novel.905/334529/30355\_1/553512/140615/550432 | 5 | 550134/30355/723999/794841\_1/795106 |
| dre00511 | Other glycan degradation | 4/900 | 28/8379 | 0.3542708 | 0.8884887 | 445250/445250\_1/566517/100000684\_1 | neu3.2/neu3.2/aga/neu3.5 | dre:445250/dre:445250/dre:566517/dre:100000684 | 4 | 1 | 566517 | 3 | 445250/445250\_1/100000684\_1 |
| dre00630 | Glyoxylate and dicarboxylate metabolism | 6/900 | 46/8379 | 0.3727921 | 0.9071011 | 335715/322670\_1/437019\_1/541329/541329\_1/792824\_1 | mdh1ab/aco2/pcca/gcsha/gcsha/pgp | dre:335715/dre:322670/dre:437019/dre:541329/dre:541329/dre:792824 | 6 | 3 | 335715/437019\_1/541329 | 3 | 322670\_1/541329\_1/792824\_1 |
| dre04510 | Focal adhesion | 40/900 | 354/8379 | 0.3903279 | 0.9071011 | 57934\_1/57934/562529\_1/30429\_2/793158/570868\_1/436718\_1/373107\_1/436718/321243\_1/563023\_1/100000252\_1/793158\_1/386856\_1/100000252\_3/100006675/30037/563023/novel.946/677750/556842\_1/571431/560564\_2/245702\_1/556226\_1/796490\_2/100005469\_1/58100\_1/378480/30429/100000252/245702/103909601/797491\_1/564565/564266/796537\_1/497384\_1/792699/101882911 | actb1/actb1/flna/mylpfa/cdc42l/itga1/rhocb/birc2/rhocb/lama5/flnb/pik3r3b/cdc42l/pdgfra/pik3r3b/prkcba/tnc/flnb/-/myl2b/tln2a/LOC571431/pdgfc/igf1rb/cdc42l2/pdgfbb/fn1a/badb/pdgfaa/mylpfa/pik3r3b/igf1rb/LOC103909601/itgb7/arhgap35/itgb3a/kdrl/prkca/rac1l/LOC101882911 | dre:57934/dre:57934/dre:562529/dre:30429/dre:793158/dre:570868/dre:436718/dre:373107/dre:436718/dre:321243/dre:563023/dre:100000252/dre:793158/dre:386856/dre:100000252/dre:100006675/dre:30037/dre:563023/dre:386856/dre:677750/dre:556842/dre:571431/dre:560564/dre:245702/dre:556226/dre:796490/dre:100005469/dre:58100/dre:103909601/dre:30429/dre:100000252/dre:245702/dre:103909601/dre:797491/dre:564565/dre:564266/dre:796537/dre:497384/dre:792699/dre:321070 | 40 | 21 | 57934\_1/562529\_1/570868\_1/436718/321243\_1/563023\_1/793158\_1/100000252\_3/100006675/30037/677750/556226\_1/796490\_2/100005469\_1/30429/100000252/245702/103909601/564565/564266/101882911 | 19 | 57934/30429\_2/793158/436718\_1/373107\_1/100000252\_1/386856\_1/563023/novel.946/556842\_1/571431/560564\_2/245702\_1/58100\_1/378480/797491\_1/796537\_1/497384\_1/792699 |
| dre00071 | Fatty acid degradation | 8/900 | 65/8379 | 0.3981676 | 0.9071011 | 100005587\_1/116517/100536795/393860\_1/368239/393860/393427/100006238\_1 | aldh9a1a.1/adh5/wu:fb14d09/gcdha/aldh2.2/gcdha/zgc:63568/aldh9a1a.2 | dre:100005587/dre:116517/dre:100536795/dre:393860/dre:368239/dre:393860/dre:393427/dre:100006238 | 8 | 3 | 116517/368239/393860 | 5 | 100005587\_1/100536795/393860\_1/393427/100006238\_1 |
| dre00860 | Porphyrin metabolism | 9/900 | 74/8379 | 0.3995793 | 0.9071011 | 100002875/100002875\_1/406731/101886332/100415796/570913\_1/100329531\_1/64607\_1/767756 | hmox2a/hmox2a/ugt1ab/hephl1b/ugt5b5/ugt5g1/hmox2b/alas2/ugt5a2 | dre:100002875/dre:100002875/dre:406731/dre:101886332/dre:678597/dre:570913/dre:100329531/dre:64607/dre:767756 | 9 | 2 | 100002875/101886332 | 7 | 100002875\_1/406731/100415796/570913\_1/100329531\_1/64607\_1/767756 |
| dre01040 | Biosynthesis of unsaturated fatty acids | 5/900 | 39/8379 | 0.4100303 | 0.9071011 | 140615/334217/393441/554145/317738\_1 | fads2/elovl7a/hacd3/elovl8b/elovl6 | dre:140615/dre:334217/dre:393441/dre:554145/dre:317738 | 5 | 3 | 140615/393441/554145 | 2 | 334217/317738\_1 |
| dre04625 | C-type lectin receptor signaling pathway | 18/900 | 156/8379 | 0.4107000 | 0.9071011 | 436718\_1/565241\_2/336121/559020/562552/436718/100000252\_1/100000252\_3/795414\_2/368241/566185/571242\_2/556277/561021/368481/565241/100000252/561021\_1 | rhocb/ppp3ccb/calm2a/ptgs2b/zgc:171775/rhocb/pik3r3b/pik3r3b/relb/egr2a/caspbl/malt3/cblb/si:dkey-103e21.5/stat1b/ppp3ccb/pik3r3b/si:dkey-103e21.5 | dre:436718/dre:565241/dre:192322/dre:559020/dre:562552/dre:436718/dre:100000252/dre:100000252/dre:795414/dre:368241/dre:566185/dre:571242/dre:556277/dre:561021/dre:368481/dre:565241/dre:100000252/dre:561021 | 18 | 11 | 336121/559020/562552/436718/100000252\_3/368241/566185/556277/561021/565241/100000252 | 7 | 436718\_1/565241\_2/100000252\_1/795414\_2/571242\_2/368481/561021\_1 |
| dre03013 | Nucleocytoplasmic transport | 15/900 | 129/8379 | 0.4130845 | 0.9071011 | 393693\_1/100005893/393693/393973/324342\_1/100005893\_1/407710\_1/406860/324342/100004503\_1/321744\_1/100004503/407710/333980/393973\_1 | sap18/magoh/sap18/rae1/xpo4/magoh/xpo7/ipo9/xpo4/eef1a1b/nup88/eef1a1b/xpo7/xpo6/rae1 | dre:393693/dre:100005893/dre:393693/dre:393973/dre:324342/dre:100005893/dre:407710/dre:406860/dre:324342/dre:100004503/dre:321744/dre:100004503/dre:407710/dre:333980/dre:393973 | 15 | 9 | 393693\_1/100005893/393973/324342\_1/406860/321744\_1/100004503/407710/333980 | 6 | 393693/100005893\_1/407710\_1/324342/100004503\_1/393973\_1 |
| dre03266 | Virion - Herpesvirus | 2/900 | 13/8379 | 0.4146866 | 0.9071011 | 791704/564266 | zgc:153759/itgb3a | dre:791704/dre:564266 | 2 | 2 | 791704/564266 | 0 |  |
| dre04330 | Notch signaling pathway | 11/900 | 93/8379 | 0.4153144 | 0.9071011 | 678649\_1/678649/566841/393500\_1/393500/557340\_1/557340/30120\_1/497616/140422\_1/140422 | zgc:136929/zgc:136929/crebbpa/zgc:66440/zgc:66440/atxn1a/atxn1a/dlc/numbl/jag2b/jag2b | dre:678649/dre:678649/dre:566841/dre:393500/dre:393500/dre:557340/dre:557340/dre:30120/dre:497616/dre:140422/dre:140422 | 11 | 7 | 678649\_1/566841/393500\_1/557340\_1/30120\_1/497616/140422 | 4 | 678649/393500/557340/140422\_1 |
| dre00480 | Glutathione metabolism | 10/900 | 85/8379 | 0.4305860 | 0.9071011 | 100330864/406762\_1/553575\_1/570579\_1/100006589\_1/555317/393493/406703/431762\_1/450084\_1 | LOC100330864/pgd/gsr/g6pd/idh1/si:ch211-276a23.5/gstt2/gsta.1/mgst1.2/gss | dre:100330864/dre:406762/dre:553575/dre:570579/dre:100006589/dre:555317/dre:393493/dre:406703/dre:431762/dre:450084 | 10 | 8 | 100330864/406762\_1/570579\_1/100006589\_1/555317/393493/431762\_1/450084\_1 | 2 | 553575\_1/406703 |
| dre00980 | Metabolism of xenobiotics by cytochrome P450 | 10/900 | 85/8379 | 0.4305860 | 0.9071011 | 116517/406731/393293/100415796/393493/570913\_1/406703/393427/431762\_1/767756 | adh5/ugt1ab/hsd11b1la/ugt5b5/gstt2/ugt5g1/gsta.1/zgc:63568/mgst1.2/ugt5a2 | dre:116517/dre:406731/dre:100006793/dre:678597/dre:393493/dre:570913/dre:406703/dre:393427/dre:431762/dre:767756 | 10 | 3 | 116517/393493/431762\_1 | 7 | 406731/393293/100415796/570913\_1/406703/393427/767756 |
| dre00982 | Drug metabolism - cytochrome P450 | 10/900 | 85/8379 | 0.4305860 | 0.9071011 | 116517/406731/100415796/393493/570913\_1/406703/393427/431762\_1/794311/767756 | adh5/ugt1ab/ugt5b5/gstt2/ugt5g1/gsta.1/zgc:63568/mgst1.2/fmo5/ugt5a2 | dre:116517/dre:406731/dre:678597/dre:393493/dre:570913/dre:406703/dre:393427/dre:431762/dre:794311/dre:767756 | 10 | 4 | 116517/393493/431762\_1/794311 | 6 | 406731/100415796/570913\_1/406703/393427/767756 |
| dre00830 | Retinol metabolism | 12/900 | 104/8379 | 0.4422248 | 0.9072910 | 116517/562840/406731/553969/445083/393580/100415796/553239/570913\_1/393427/407663/767756 | adh5/rdh10a/ugt1ab/cyp3a65/dhrs3a/bco1l/ugt5b5/lrata/ugt5g1/zgc:63568/si:ch211-107o10.3/ugt5a2 | dre:116517/dre:562840/dre:406731/dre:553969/dre:445083/dre:393580/dre:678597/dre:553239/dre:570913/dre:393427/dre:407663/dre:767756 | 12 | 5 | 116517/562840/553969/445083/393580 | 7 | 406731/100415796/553239/570913\_1/393427/407663/767756 |
| dre04621 | NOD-like receptor signaling pathway | 23/900 | 205/8379 | 0.4451656 | 0.9072910 | 100000242/436718\_1/373107\_1/393186\_1/562552/436718/557239/326974\_1/436734\_1/569298/566185/564295\_2/557239\_1/561021/323944/100334992/368481/110437957/100002946/110438502/561021\_1/403131/100149641 | zgc:92606/rhocb/birc2/zgc:56235/zgc:171775/rhocb/antxr2a/gabarapa/txn/ctsbb/caspbl/traf2a/antxr2a/si:dkey-103e21.5/si:ch1073-155h21.1/unm\_sa911/stat1b/LOC110437957/cxcl8a/LOC110438502/si:dkey-103e21.5/tlr4ba/LOC100149641 | dre:100000242/dre:436718/dre:373107/dre:393186/dre:562552/dre:436718/dre:557239/dre:326974/dre:436734/dre:569298/dre:566185/dre:564295/dre:557239/dre:561021/dre:323944/dre:100334992/dre:368481/dre:373874/dre:100002946/dre:796505/dre:561021/dre:403131/dre:100149641 | 23 | 10 | 393186\_1/562552/436718/326974\_1/569298/566185/557239\_1/561021/110437957/110438502 | 13 | 100000242/436718\_1/373107\_1/557239/436734\_1/564295\_2/323944/100334992/368481/100002946/561021\_1/403131/100149641 |
| dre04217 | Necroptosis | 24/900 | 215/8379 | 0.4529389 | 0.9072910 | 336426\_1/559768/321622/110438871/393444/373107\_1/336426/393186\_1/337413/337413\_1/550542\_1/558816/100126128/566185/564295\_2/768248/561021/368481/100148818/100149436\_1/100149436/561021\_1/100148591/403131 | chmp1b/zgc:173593/hmgb1a/LOC110438871/pygmb/birc2/chmp1b/zgc:56235/stat6/stat6/chmp4c/LOC558816/zgc:173594/caspbl/traf2a/fas/si:dkey-103e21.5/stat1b/si:ch73-55i23.1/si:ch211-189a15.5/si:ch211-189a15.5/si:dkey-103e21.5/LOC100148591/tlr4ba | dre:336426/dre:559768/dre:110438871/dre:110438871/dre:393444/dre:373107/dre:336426/dre:393186/dre:337413/dre:337413/dre:550542/dre:558816/dre:100126128/dre:566185/dre:564295/dre:768248/dre:561021/dre:368481/dre:100148818/dre:100149436/dre:100149436/dre:561021/dre:561187/dre:403131 | 24 | 10 | 110438871/393444/336426/393186\_1/337413\_1/566185/768248/561021/100149436/100148591 | 14 | 336426\_1/559768/321622/373107\_1/337413/550542\_1/558816/100126128/564295\_2/368481/100148818/100149436\_1/561021\_1/403131 |
| dre04146 | Peroxisome | 13/900 | 114/8379 | 0.4536455 | 0.9072910 | 100093708\_1/450048/100006589\_1/406803/562105/100093708/405800/406803\_1/406803\_2/100003383\_1/100003383/110440136/619259 | slc25a17/ech1/idh1/abcd3a/si:dkey-275b16.2/slc25a17/dao.2/abcd3a/abcd3a/pmvk/pmvk/LOC110440136/dao.1 | dre:100093708/dre:450048/dre:100006589/dre:406803/dre:110440136/dre:100093708/dre:405800/dre:406803/dre:406803/dre:100003383/dre:100003383/dre:110440136/dre:619259 | 13 | 7 | 100093708\_1/100006589\_1/405800/406803\_1/406803\_2/100003383/619259 | 6 | 450048/406803/562105/100093708/100003383\_1/110440136 |
| dre00910 | Nitrogen metabolism | 3/900 | 24/8379 | 0.4844481 | 0.9567850 | 569989\_1/100006448/767680 | ca5a/ca6/zgc:153760 | dre:569989/dre:100006448/dre:767680 | 3 | 2 | 569989\_1/767680 | 1 | 100006448 |
| dre04068 | FoxO signaling pathway | 23/900 | 210/8379 | 0.4938151 | 0.9585940 | 324140\_1/324140/100000242/406473/431763\_1/117508\_1/431763/566841/562552/100000252\_1/117508/767751\_1/100000252\_3/326974\_1/571431/245702\_1/565576/563708/100000252/245702/100006858\_1/280649\_1/559723 | sgk1/sgk1/zgc:92606/pck2/gadd45ab/klf2a/gadd45ab/crebbpa/zgc:171775/pik3r3b/klf2a/ccnb3/pik3r3b/gabarapa/LOC571431/igf1rb/plk2b/skp2/pik3r3b/igf1rb/csnk1e/plk1/tgfb2l | dre:324140/dre:324140/dre:100000242/dre:406473/dre:431763/dre:117508/dre:431763/dre:566841/dre:562552/dre:100000252/dre:117508/dre:767751/dre:100000252/dre:326974/dre:571431/dre:245702/dre:565576/dre:563708/dre:100000252/dre:245702/dre:100006858/dre:280649/dre:559723 | 23 | 15 | 324140\_1/406473/117508\_1/431763/566841/562552/117508/767751\_1/100000252\_3/326974\_1/565576/100000252/245702/280649\_1/559723 | 8 | 324140/100000242/431763\_1/100000252\_1/571431/245702\_1/563708/100006858\_1 |
| dre00280 | Valine, leucine and isoleucine degradation | 8/900 | 71/8379 | 0.4992625 | 0.9585940 | 100005587\_1/100536795/368239/437019\_1/394130\_1/797311/777632/100006238\_1 | aldh9a1a.1/wu:fb14d09/aldh2.2/pcca/acad8/LOC797311/mccc1/aldh9a1a.2 | dre:100005587/dre:100536795/dre:368239/dre:437019/dre:394130/dre:797311/dre:777632/dre:100006238 | 8 | 5 | 368239/437019\_1/394130\_1/797311/777632 | 3 | 100005587\_1/100536795/100006238\_1 |
| dre00040 | Pentose and glucuronate interconversions | 7/900 | 62/8379 | 0.5045854 | 0.9585940 | 406731/100415796/570913\_1/445326\_1/393350/445326/767756 | ugt1ab/ugt5b5/ugt5g1/akr1a1a/xylb/akr1a1a/ugt5a2 | dre:406731/dre:678597/dre:570913/dre:445326/dre:393350/dre:445326/dre:767756 | 7 | 2 | 445326\_1/393350 | 5 | 406731/100415796/570913\_1/445326/767756 |
| dre00670 | One carbon pool by folate | 3/900 | 25/8379 | 0.5125722 | 0.9585940 | 100526683\_1/557552/100004977\_2 | aldh1l1/zgc:153031/mthfd2 | dre:100526683/dre:557552/dre:100004977 | 3 | 1 | 557552 | 2 | 100526683\_1/100004977\_2 |
| dre04623 | Cytosolic DNA-sensing pathway | 8/900 | 72/8379 | 0.5156993 | 0.9585940 | 140621/394162/562329/566185/394162\_1/140621\_1/561021/561021\_1 | casp3a/polr3f/polr3b/caspbl/polr3f/casp3a/si:dkey-103e21.5/si:dkey-103e21.5 | dre:140621/dre:394162/dre:562329/dre:566185/dre:394162/dre:140621/dre:561021/dre:561021 | 8 | 4 | 562329/566185/394162\_1/561021 | 4 | 140621/394162/140621\_1/561021\_1 |
| dre04216 | Ferroptosis | 8/900 | 73/8379 | 0.5319511 | 0.9773055 | 559768/796322/393186\_1/558816/100126128/790938/450084\_1/100320152 | zgc:173593/slc3a2a/zgc:56235/LOC558816/zgc:173594/zgc:158423/gss/steap3 | dre:559768/dre:796322/dre:393186/dre:558816/dre:100126128/dre:790938/dre:450084/dre:100320152 | 8 | 3 | 796322/393186\_1/450084\_1 | 5 | 559768/558816/100126128/790938/100320152 |
| dre04530 | Tight junction | 32/900 | 300/8379 | 0.5455885 | 0.9807608 | 57934\_1/337226\_1/337226/57934/286739\_1/569084\_1/793158/436718\_1/286739/81585/792828/436718/60635\_1/798632/793158\_1/100536545/792828\_1/110438642/677750/556226\_1/793143/556298/797651/797651\_1/559392\_1/431777/445305\_1/793952\_1/100005566/559392/792699/101882911 | actb1/zgc:153867/zgc:153867/actb1/msna/cldni/cdc42l/rhocb/msna/cldnf/pard6a/rhocb/cldn7b/myl6/cdc42l/synpo/pard6a/LOC110438642/myl2b/cdc42l2/si:dkey-98f17.3/micall2b/jam3a/jam3a/ppp2r2cb/tuba7l/arpc4l/si:dkey-253i9.4/f11r.2/ppp2r2cb/rac1l/LOC101882911 | dre:57934/dre:337226/dre:337226/dre:57934/dre:286739/dre:569084/dre:793158/dre:436718/dre:286739/dre:81585/dre:792828/dre:436718/dre:60635/dre:798632/dre:793158/dre:100536545/dre:792828/dre:110438642/dre:677750/dre:556226/dre:793143/dre:556298/dre:797651/dre:797651/dre:559392/dre:431777/dre:445305/dre:793952/dre:100005566/dre:559392/dre:792699/dre:321070 | 32 | 16 | 57934\_1/337226\_1/286739/436718/798632/793158\_1/792828\_1/677750/556226\_1/793143/797651\_1/559392\_1/431777/793952\_1/100005566/101882911 | 16 | 337226/57934/286739\_1/569084\_1/793158/436718\_1/81585/792828/60635\_1/100536545/110438642/556298/797651/445305\_1/559392/792699 |
| dre00514 | Other types of O-glycan biosynthesis | 7/900 | 65/8379 | 0.5564938 | 0.9807608 | 556077\_1/557675/557675\_1/555344/569769/337131/568500 | plod3/c1galt1a/c1galt1a/si:dkey-202e17.1/pomt1/c1galt1b/galnt8a.2 | dre:556077/dre:557675/dre:557675/dre:555344/dre:569769/dre:337131/dre:568500 | 7 | 4 | 556077\_1/557675\_1/555344/337131 | 3 | 557675/569769/568500 |
| dre04540 | Gap junction | 17/900 | 160/8379 | 0.5575795 | 0.9807608 | 323509\_1/386856\_1/406811\_1/100006675/568726/novel.946/571431/560564\_2/796490\_2/378480/100000981\_2/566430\_1/431777/560807/103909601/497384\_1/559806 | gnaia/pdgfra/zgc:55461/prkcba/adcy7/-/LOC571431/pdgfc/pdgfbb/pdgfaa/htr2cl1/prkg1b/tuba7l/adcy6b/LOC103909601/prkca/htr2ab | dre:323509/dre:386856/dre:336681/dre:100006675/dre:568726/dre:386856/dre:571431/dre:560564/dre:796490/dre:103909601/dre:100000981/dre:566430/dre:431777/dre:560807/dre:103909601/dre:497384/dre:559806 | 17 | 8 | 323509\_1/100006675/568726/796490\_2/431777/560807/103909601/559806 | 9 | 386856\_1/406811\_1/novel.946/571431/560564\_2/378480/100000981\_2/566430\_1/497384\_1 |
| dre00062 | Fatty acid elongation | 5/900 | 46/8379 | 0.5590536 | 0.9807608 | 334217/393441/554145/393657/317738\_1 | elovl7a/hacd3/elovl8b/zgc:66024/elovl6 | dre:334217/dre:393441/dre:554145/dre:393657/dre:317738 | 5 | 3 | 393441/554145/393657 | 2 | 334217/317738\_1 |
| dre04916 | Melanogenesis | 18/900 | 171/8379 | 0.5737918 | 0.9807608 | 323509\_1/336121/566841/100006675/568726/100333318/556087\_1/58072/30369\_1/791993/393760\_1/30127\_1/560807/100538072/556087/553181\_1/58072\_1/497384\_1 | gnaia/calm2a/crebbpa/prkcba/adcy7/creb3l4/wnt2bb/fzd8b/fzd7a/wnt4b/gnao1a/wnt2/adcy6b/creb3l3b/wnt2bb/kitlgb/fzd8b/prkca | dre:323509/dre:192322/dre:566841/dre:100006675/dre:568726/dre:100333318/dre:556087/dre:58072/dre:30369/dre:791993/dre:393760/dre:30127/dre:560807/dre:100538072/dre:556087/dre:553181/dre:58072/dre:497384 | 18 | 10 | 323509\_1/336121/566841/100006675/568726/560807/100538072/556087/553181\_1/58072\_1 | 8 | 100333318/556087\_1/58072/30369\_1/791993/393760\_1/30127\_1/497384\_1 |
| dre00250 | Alanine, aspartate and glutamate metabolism | 6/900 | 57/8379 | 0.5844578 | 0.9807608 | 447815\_1/334431\_1/447815/564754\_1/565235\_1/406688 | rimkla/adsl/rimkla/nat8l/aldh5a1/got2a | dre:447815/dre:334431/dre:447815/dre:564754/dre:565235/dre:406688 | 6 | 3 | 334431\_1/447815/565235\_1 | 3 | 447815\_1/564754\_1/406688 |
| dre00730 | Thiamine metabolism | 2/900 | 18/8379 | 0.5907726 | 0.9807608 | 100330410/574009 | alp3/alpi.2 | dre:100330410/dre:574009 | 2 | 2 | 100330410/574009 | 0 |  |
| dre00770 | Pantothenate and CoA biosynthesis | 3/900 | 28/8379 | 0.5916833 | 0.9807608 | 100536795/368239/406789 | wu:fb14d09/aldh2.2/pank1a | dre:100536795/dre:368239/dre:406789 | 3 | 2 | 368239/406789 | 1 | 100536795 |
| dre00790 | Folate biosynthesis | 4/900 | 38/8379 | 0.5945768 | 0.9807608 | 557552/791448\_1/100330410/574009 | zgc:153031/qdprb1/alp3/alpi.2 | dre:557552/dre:791448/dre:100330410/dre:574009 | 4 | 4 | 557552/791448\_1/100330410/574009 | 0 |  |
| dre04520 | Adherens junction | 20/900 | 194/8379 | 0.6129104 | 0.9807608 | 57934\_1/57934/30429\_2/793158/436718\_1/566841/436718/793158\_1/677750/569566/335573\_1/571431/245702\_1/556226\_1/393527/30429/245702/564805\_1/792699/101882911 | actb1/actb1/mylpfa/cdc42l/rhocb/crebbpa/rhocb/cdc42l/myl2b/LOC569566/ptpn6/LOC571431/igf1rb/cdc42l2/pdcd10a/mylpfa/igf1rb/arhgap29a/rac1l/LOC101882911 | dre:57934/dre:57934/dre:30429/dre:793158/dre:436718/dre:566841/dre:436718/dre:793158/dre:677750/dre:569566/dre:335573/dre:571431/dre:245702/dre:556226/dre:393527/dre:30429/dre:245702/dre:378998/dre:792699/dre:321070 | 20 | 12 | 57934\_1/566841/436718/793158\_1/677750/335573\_1/556226\_1/393527/30429/245702/564805\_1/101882911 | 8 | 57934/30429\_2/793158/436718\_1/569566/571431/245702\_1/792699 |
| dre04142 | Lysosome | 22/900 | 214/8379 | 0.6206336 | 0.9807608 | 565099/571066/100003043\_1/406511/553423\_1/569298/337771/450083/368911/436725\_1/553423/566517/445244/436725/559575/565246/327237\_1/570148\_1/406511\_1/393657/445244\_1/567720 | zgc:193725/si:ch1073-513e17.1/lamp3/sort1a/sumf1/ctsbb/zgc:162858/gla/laptm4b/acp5b/sumf1/aga/ap1m3/acp5b/si:ch211-15d5.11/si:dkey-192p21.6/ap1s2/abcb9/sort1a/zgc:66024/ap1m3/idua | dre:565099/dre:571066/dre:100003043/dre:406511/dre:553423/dre:569298/dre:337771/dre:450083/dre:368911/dre:436725/dre:553423/dre:566517/dre:445244/dre:436725/dre:559575/dre:565246/dre:327237/dre:570148/dre:406511/dre:393657/dre:445244/dre:567720 | 22 | 15 | 565099/100003043\_1/569298/450083/368911/436725\_1/553423/566517/445244/559575/327237\_1/570148\_1/406511\_1/393657/567720 | 7 | 571066/406511/553423\_1/337771/436725/565246/445244\_1 |
| dre00604 | Glycosphingolipid biosynthesis - ganglio series | 2/900 | 19/8379 | 0.6208978 | 0.9807608 | 394230/394230\_1 | st3gal5/st3gal5 | dre:394230/dre:394230 | 2 | 1 | 394230 | 1 | 394230\_1 |
| dre04261 | Adrenergic signaling in cardiomyocytes | 31/900 | 301/8379 | 0.6279826 | 0.9807608 | 323509\_1/336121/64614/562552/556410\_1/793666\_1/100535196/568726/555423\_1/677750/100333318/100037358\_1/793666/559015/794522/678560\_1/562282\_1/555423/559392\_1/562282/492486\_1/114457\_1/353247/550357\_2/560807/492486/100538072/114457/559392/558525\_1/497384\_1 | gnaia/calm2a/atp1a1a.3/zgc:171775/atf4b/popdc2/cacna2d3a/adcy7/scn1ba/myl2b/creb3l4/zgc:163073/popdc2/cacnb3b/si:ch211-139a5.9/cacnb4b/ppp2r5b/scn1ba/ppp2r2cb/ppp2r5b/adra1bb/atp1b2b/tnnc1a/cremb/adcy6b/adra1bb/creb3l3b/atp1b2b/ppp2r2cb/atp2b3b/prkca | dre:323509/dre:192322/dre:64614/dre:562552/dre:556410/dre:793666/dre:100535196/dre:568726/dre:555423/dre:677750/dre:100333318/dre:100037358/dre:793666/dre:559015/dre:794522/dre:678560/dre:562282/dre:555423/dre:559392/dre:562282/dre:492486/dre:114457/dre:353247/dre:550357/dre:560807/dre:492486/dre:100538072/dre:114457/dre:559392/dre:558525/dre:497384 | 31 | 17 | 323509\_1/336121/562552/556410\_1/793666\_1/100535196/568726/555423\_1/677750/794522/559392\_1/562282/114457\_1/560807/492486/100538072/558525\_1 | 14 | 64614/100333318/100037358\_1/793666/559015/678560\_1/562282\_1/555423/492486\_1/353247/550357\_2/114457/559392/497384\_1 |
| dre04912 | GnRH signaling pathway | 16/900 | 158/8379 | 0.6379224 | 0.9807608 | 793158/336121/30498\_1/562552/556410\_1/793158\_1/566945/100006675/30498/568726/571431/556226\_1/565743\_1/100148818/560807/497384\_1 | cdc42l/calm2a/egr1/zgc:171775/atf4b/cdc42l/mmp14b/prkcba/egr1/adcy7/LOC571431/cdc42l2/pld2/si:ch73-55i23.1/adcy6b/prkca | dre:793158/dre:192322/dre:30498/dre:562552/dre:556410/dre:793158/dre:566945/dre:100006675/dre:30498/dre:568726/dre:571431/dre:556226/dre:565743/dre:100148818/dre:560807/dre:497384 | 16 | 10 | 336121/30498\_1/562552/556410\_1/793158\_1/100006675/568726/556226\_1/565743\_1/560807 | 6 | 793158/566945/30498/571431/100148818/497384\_1 |
| dre04270 | Vascular smooth muscle contraction | 23/900 | 226/8379 | 0.6417536 | 0.9807608 | 337226\_1/337226/436718\_1/336121/406370\_1/436718/798632/406370/100006675/436966\_2/568726/403114\_1/567131/436966/403114/492486\_1/566430\_1/100148818/393783\_1/560807/492486/497384\_1/101882911 | zgc:153867/zgc:153867/rhocb/calm2a/pla2g6/rhocb/myl6/pla2g6/prkcba/zgc:91818/adcy7/adm2a/calcrlb/zgc:91818/adm2a/adra1bb/prkg1b/si:ch73-55i23.1/ppp1r14ab/adcy6b/adra1bb/prkca/LOC101882911 | dre:337226/dre:337226/dre:436718/dre:192322/dre:406370/dre:436718/dre:798632/dre:406370/dre:100006675/dre:436966/dre:568726/dre:403114/dre:567131/dre:436966/dre:403114/dre:492486/dre:566430/dre:100148818/dre:393783/dre:560807/dre:492486/dre:497384/dre:321070 | 23 | 14 | 337226\_1/336121/436718/798632/406370/100006675/436966\_2/568726/403114\_1/567131/393783\_1/560807/492486/101882911 | 9 | 337226/436718\_1/406370\_1/436966/403114/492486\_1/566430\_1/100148818/497384\_1 |
| dre01250 | Biosynthesis of nucleotide sugars | 5/900 | 51/8379 | 0.6529599 | 0.9807608 | 406339\_1/100007686/100885772/321224/751668 | hk2/LOC100007686/cmasb/hkdc1/gck | dre:406339/dre:100007686/dre:100885772/dre:321224/dre:751668 | 5 | 3 | 100885772/321224/751668 | 2 | 406339\_1/100007686 |
| dre04136 | Autophagy - other | 4/900 | 41/8379 | 0.6557403 | 0.9807608 | 100000242/792689\_1/326974\_1/406843\_1 | zgc:92606/mlst8/gabarapa/igbp1 | dre:100000242/dre:792689/dre:326974/dre:406843 | 4 | 2 | 326974\_1/406843\_1 | 2 | 100000242/792689\_1 |
| dre04620 | Toll-like receptor signaling pathway | 12/900 | 121/8379 | 0.6596097 | 0.9807608 | 562552/100000252\_1/100000252\_3/394198/570238/561021/368481/100002946/100000252/561021\_1/403131/792699 | zgc:171775/pik3r3b/pik3r3b/fosab/fosaa/si:dkey-103e21.5/stat1b/cxcl8a/pik3r3b/si:dkey-103e21.5/tlr4ba/rac1l | dre:562552/dre:100000252/dre:100000252/dre:394198/dre:570238/dre:561021/dre:368481/dre:100002946/dre:100000252/dre:561021/dre:403131/dre:792699 | 12 | 6 | 562552/100000252\_3/394198/570238/561021/100000252 | 6 | 100000252\_1/368481/100002946/561021\_1/403131/792699 |
| dre04145 | Phagosome | 21/900 | 209/8379 | 0.6619298 | 0.9807608 | 57934\_1/57934/359840\_1/406811\_1/359840/100004793/30762/571584/368878/100537272/403014/100005358/30763/431777/359839/571282/403131/564266/556826\_1/792699/100007982 | actb1/actb1/atp6v1b2/zgc:55461/atp6v1b2/mrc2/mhc2dab/marco/si:busm1-266f07.2/LOC100537272/ncf4/si:ch211-274f20.2/mhc2dcb/tuba7l/atp6v1ba/LOC571282/tlr4ba/itgb3a/si:dkey-57h18.2/rac1l/hbl1 | dre:57934/dre:57934/dre:359840/dre:336681/dre:359840/dre:100004793/dre:30762/dre:571584/dre:368878/dre:100537272/dre:403014/dre:100005358/dre:30763/dre:431777/dre:359839/dre:571282/dre:403131/dre:564266/dre:556826/dre:792699/dre:100007982 | 21 | 13 | 57934\_1/359840/100004793/30762/368878/100537272/403014/30763/431777/359839/571282/564266/556826\_1 | 8 | 57934/359840\_1/406811\_1/571584/100005358/403131/792699/100007982 |
| dre00120 | Primary bile acid biosynthesis | 3/900 | 31/8379 | 0.6619931 | 0.9807608 | 723999/641477/795106 | zgc:136333/zgc:123299/cyp27a1.2 | dre:795106/dre:553543/dre:795106 | 3 | 1 | 641477 | 2 | 723999/795106 |
| dre03020 | RNA polymerase | 4/900 | 42/8379 | 0.6746907 | 0.9807608 | 394162/325603/562329/394162\_1 | polr3f/polr1b/polr3b/polr3f | dre:394162/dre:325603/dre:562329/dre:394162 | 4 | 2 | 562329/394162\_1 | 2 | 394162/325603 |
| dre04914 | Progesterone-mediated oocyte maturation | 13/900 | 133/8379 | 0.6831474 | 0.9807608 | 323509\_1/562552/100000252\_1/767751\_1/100000252\_3/568726/245702\_1/100000252/393223\_1/560807/245702/100009657\_1/280649\_1 | gnaia/zgc:171775/pik3r3b/ccnb3/pik3r3b/adcy7/igf1rb/pik3r3b/fzr1a/adcy6b/igf1rb/rps6ka3b/plk1 | dre:323509/dre:562552/dre:100000252/dre:767751/dre:100000252/dre:568726/dre:245702/dre:100000252/dre:393223/dre:560807/dre:245702/dre:100009657/dre:280649 | 13 | 10 | 323509\_1/562552/767751\_1/100000252\_3/568726/100000252/560807/245702/100009657\_1/280649\_1 | 3 | 100000252\_1/245702\_1/393223\_1 |
| dre04140 | Autophagy - animal | 24/900 | 241/8379 | 0.6857514 | 0.9807608 | 321622/100000242/110438871/792229\_1/792229/449923/100000252\_1/573492/792689\_1/100000252\_3/326974\_1/567021/449923\_1/569298/245702\_1/100330617\_1/58100\_1/559564/563140/406843\_1/563140\_1/100000252/245702/573492\_1 | hmgb1a/zgc:92606/LOC110438871/sh3glb1a/sh3glb1a/si:ch73-234b20.5/pik3r3b/rragca/mlst8/pik3r3b/gabarapa/ern2/si:ch73-234b20.5/ctsbb/igf1rb/tsc1b/badb/eif2ak3/si:ch211-11n16.2/igbp1/si:ch211-11n16.2/pik3r3b/igf1rb/rragca | dre:110438871/dre:100000242/dre:110438871/dre:792229/dre:792229/dre:449923/dre:100000252/dre:573492/dre:792689/dre:100000252/dre:326974/dre:567021/dre:449923/dre:569298/dre:245702/dre:100330617/dre:58100/dre:559564/dre:563140/dre:406843/dre:563140/dre:100000252/dre:245702/dre:573492 | 24 | 14 | 110438871/792229\_1/449923/100000252\_3/326974\_1/567021/449923\_1/569298/100330617\_1/563140/406843\_1/100000252/245702/573492\_1 | 10 | 321622/100000242/792229/100000252\_1/573492/792689\_1/245702\_1/58100\_1/559564/563140\_1 |
| dre04514 | Cell adhesion molecules | 20/900 | 204/8379 | 0.7025826 | 0.9807608 | 569084\_1/30194/81585/60635\_1/557935/110438642/30762/793143/30634\_1/368878/797651/797651\_1/30763/571282/101886406/541474/797491\_1/100151365\_1/100005566/562934\_1 | cldni/alcama/cldnf/cldn7b/zmp:0000001082/LOC110438642/mhc2dab/si:dkey-98f17.3/nadl1.2/si:busm1-266f07.2/jam3a/jam3a/mhc2dcb/LOC571282/cd28/cntn1b/itgb7/si:dkey-1h24.6/f11r.2/esamb | dre:569084/dre:30194/dre:81585/dre:60635/dre:557935/dre:110438642/dre:30762/dre:793143/dre:30634/dre:368878/dre:797651/dre:797651/dre:30763/dre:571282/dre:101886406/dre:541474/dre:797491/dre:100151365/dre:100005566/dre:562934 | 20 | 12 | 30194/30762/793143/30634\_1/368878/797651\_1/30763/571282/541474/100151365\_1/100005566/562934\_1 | 8 | 569084\_1/81585/60635\_1/557935/110438642/797651/101886406/797491\_1 |
| dre00564 | Glycerophospholipid metabolism | 13/900 | 135/8379 | 0.7038353 | 0.9807608 | 724010/100002015\_1/724010\_1/406370\_1/406370/799099\_1/334017\_1/565743\_1/560225/560649/767688/100148818/572841 | dgkaa/cds1/dgkaa/pla2g6/pla2g6/lpin1/pla1a/pld2/cept1a/si:ch211-214p16.3/zgc:154040/si:ch73-55i23.1/mboat1 | dre:724010/dre:100002015/dre:724010/dre:406370/dre:406370/dre:799099/dre:334017/dre:565743/dre:560225/dre:560649/dre:767688/dre:100148818/dre:572841 | 13 | 9 | 100002015\_1/724010\_1/406370/799099\_1/334017\_1/565743\_1/560225/560649/572841 | 4 | 724010/406370\_1/767688/100148818 |
| dre04622 | RIG-I-like receptor signaling pathway | 7/900 | 76/8379 | 0.7217318 | 0.9807608 | 562552/564295\_2/561021/100002946/393144\_2/100148871/561021\_1 | zgc:171775/traf2a/si:dkey-103e21.5/cxcl8a/trim25/dhx58/si:dkey-103e21.5 | dre:562552/dre:564295/dre:561021/dre:100002946/dre:393144/dre:100148871/dre:561021 | 7 | 4 | 562552/561021/393144\_2/100148871 | 3 | 564295\_2/100002946/561021\_1 |
| dre03022 | Basal transcription factors | 4/900 | 45/8379 | 0.7271209 | 0.9807608 | 798997\_2/798997\_1/567899/798997 | gtf2a2/gtf2a2/gtf2e1/gtf2a2 | dre:798997/dre:798997/dre:567899/dre:798997 | 4 | 2 | 798997\_1/798997 | 2 | 798997\_2/567899 |
| dre04310 | Wnt signaling pathway | 27/900 | 278/8379 | 0.7415847 | 0.9807608 | 436718\_1/565241\_2/678649\_1/678649/566841/436718/323883/100000500\_1/100006675/556087\_1/58072/30369\_1/564241/103909653/791993/565241/402843\_1/30127\_1/30197/556087/58072\_1/100006858\_1/798564/326875\_1/497384\_1/792699/101882911 | rhocb/ppp3ccb/zgc:136929/zgc:136929/crebbpa/rhocb/cul1a/sost/prkcba/wnt2bb/fzd8b/fzd7a/fosl1a/LOC103909653/wnt4b/ppp3ccb/sfrp1a/wnt2/dkk1b/wnt2bb/fzd8b/csnk1e/sfrp1b/cby1/prkca/rac1l/LOC101882911 | dre:436718/dre:565241/dre:678649/dre:678649/dre:566841/dre:436718/dre:323883/dre:100000500/dre:100006675/dre:556087/dre:58072/dre:30369/dre:564241/dre:103909653/dre:791993/dre:565241/dre:402843/dre:30127/dre:30197/dre:556087/dre:58072/dre:100006858/dre:798564/dre:326875/dre:497384/dre:792699/dre:321070 | 27 | 11 | 678649\_1/566841/436718/100000500\_1/100006675/564241/103909653/565241/556087/58072\_1/101882911 | 16 | 436718\_1/565241\_2/678649/323883/556087\_1/58072/30369\_1/791993/402843\_1/30127\_1/30197/100006858\_1/798564/326875\_1/497384\_1/792699 |
| dre03420 | Nucleotide excision repair | 6/900 | 68/8379 | 0.7527347 | 0.9807608 | 393774\_2/393774/556995\_1/556995\_2/553405\_1/100006257 | pole3/pole3/lig1/lig1/pole/cetn2 | dre:393774/dre:393774/dre:556995/dre:556995/dre:553405/dre:100006257 | 6 | 3 | 393774/556995\_1/100006257 | 3 | 393774\_2/556995\_2/553405\_1 |
| dre04910 | Insulin signaling pathway | 20/900 | 212/8379 | 0.7648492 | 0.9807608 | 100001260\_1/393444/406473/336121/100001260/79380/100000252\_1/79380\_1/100000252\_3/406339\_1/556277/100330617\_1/58100\_1/559403\_1/100000252/559001\_1/321224/751668/406459/569706\_1 | ppp1r3ab/pygmb/pck2/calm2a/ppp1r3ab/eif4ea/pik3r3b/eif4ea/pik3r3b/hk2/cblb/tsc1b/badb/acaca/pik3r3b/fasn/hkdc1/gck/zgc:77112/phkb | dre:100001260/dre:393444/dre:406473/dre:192322/dre:100001260/dre:79380/dre:100000252/dre:79380/dre:100000252/dre:406339/dre:556277/dre:100330617/dre:58100/dre:559403/dre:100000252/dre:559001/dre:321224/dre:751668/dre:406459/dre:569706 | 20 | 14 | 100001260\_1/393444/406473/336121/79380/100000252\_3/556277/100330617\_1/559403\_1/100000252/321224/751668/406459/569706\_1 | 6 | 100001260/100000252\_1/79380\_1/406339\_1/58100\_1/559001\_1 |
| dre04060 | Cytokine-cytokine receptor interaction | 23/900 | 242/8379 | 0.7654644 | 0.9807608 | 799354/368326\_1/58151\_1/561050\_1/799279/368438\_1/100037357/799354\_1/368326/562247/768248/100006078\_1/100135755/562247\_1/58133/100002946/791704/100006078/100135062\_2/100001424/100333914/30125/559723 | cxcl12b/il13ra2/cxcl14/ackr3b/LOC799279/cntfr/tnfrsf1b/cxcl12b/il13ra2/clcf1/fas/ccr9b/il6r/clcf1/ngfb/cxcl8a/zgc:153759/ccr9b/csf2rb/ghra/ccl25a/gdf3/tgfb2l | dre:799354/dre:368326/dre:58151/dre:561050/dre:799279/dre:368438/dre:100037357/dre:799354/dre:368326/dre:562247/dre:768248/dre:100006078/dre:100135755/dre:562247/dre:58133/dre:100002946/dre:791704/dre:100006078/dre:100135062/dre:100001424/dre:100333914/dre:30125/dre:559723 | 23 | 17 | 799354/368326\_1/58151\_1/561050\_1/799279/368438\_1/562247/768248/100135755/58133/791704/100006078/100135062\_2/100001424/100333914/30125/559723 | 6 | 100037357/799354\_1/368326/100006078\_1/562247\_1/100002946 |
| dre00563 | Glycosylphosphatidylinositol (GPI)-anchor biosynthesis | 2/900 | 25/8379 | 0.7664229 | 0.9807608 | 561471/561471\_1 | pigl/pigl | dre:561471/dre:561471 | 2 | 1 | 561471\_1 | 1 | 561471 |
| dre04020 | Calcium signaling pathway | 44/900 | 450/8379 | 0.7730399 | 0.9807608 | 565241\_2/336121/393186\_1/368717/368717\_1/544664/386856\_1/544664\_1/100006675/568726/566671/100005049/novel.946/569566/571431/560564\_2/387296\_1/796490\_2/494456/58147/58147\_1/378480/492486\_1/100000981\_2/101886891/565241/58133/353247/259258/492486/794365/100005590/103909601/445226/558525\_1/796537\_1/100005590\_1/497384\_1/555941/559806/100137117/569706\_1/570493\_1/100137117\_1 | ppp3ccb/calm2a/zgc:56235/itpka/itpka/erbb3b/pdgfra/erbb3b/prkcba/adcy7/orai1a/fgf1b/-/LOC569566/LOC571431/pdgfc/p2rx1/pdgfbb/fgf23/p2rx3a/p2rx3a/pdgfaa/adra1bb/htr2cl1/LOC101886891/ppp3ccb/ngfb/tnnc1a/p2rx4a/adra1bb/si:dkey-163m14.7/hrh2b/LOC103909601/casq1a/atp2b3b/kdrl/hrh2b/prkca/cacna1ba/htr2ab/si:dkey-1h24.2/phkb/grin2ab/si:dkey-1h24.2 | dre:565241/dre:192322/dre:393186/dre:368717/dre:368717/dre:544664/dre:386856/dre:100149258/dre:100006675/dre:568726/dre:566671/dre:100005049/dre:386856/dre:569566/dre:571431/dre:560564/dre:387296/dre:796490/dre:494456/dre:58147/dre:58147/dre:103909601/dre:492486/dre:100000981/dre:101886891/dre:565241/dre:58133/dre:353247/dre:259258/dre:492486/dre:794365/dre:100005590/dre:103909601/dre:445226/dre:558525/dre:796537/dre:100005590/dre:497384/dre:555941/dre:559806/dre:100137117/dre:569706/dre:570493/dre:100137117 | 44 | 22 | 336121/393186\_1/368717/544664\_1/100006675/568726/796490\_2/494456/58147/101886891/565241/58133/492486/794365/100005590/103909601/445226/558525\_1/555941/559806/569706\_1/100137117\_1 | 22 | 565241\_2/368717\_1/544664/386856\_1/566671/100005049/novel.946/569566/571431/560564\_2/387296\_1/58147\_1/378480/492486\_1/100000981\_2/353247/259258/796537\_1/100005590\_1/497384\_1/100137117/570493\_1 |
| dre04260 | Cardiac muscle contraction | 16/900 | 173/8379 | 0.7739023 | 0.9807608 | 393793\_1/64614/447942\_1/100535196/447942/677750/556638\_2/100037358\_1/559015/794522/678560\_1/114457\_1/353247/556638\_1/114457/436768 | uqcrc1/atp1a1a.3/cox6a2/cacna2d3a/cox6a2/myl2b/slc9a6b/zgc:163073/cacnb3b/si:ch211-139a5.9/cacnb4b/atp1b2b/tnnc1a/slc9a6b/atp1b2b/uqcrq | dre:393793/dre:64614/dre:447942/dre:100535196/dre:447942/dre:677750/dre:556638/dre:100037358/dre:559015/dre:794522/dre:678560/dre:114457/dre:353247/dre:556638/dre:114457/dre:436768 | 16 | 6 | 100535196/447942/677750/556638\_2/794522/114457\_1 | 10 | 393793\_1/64614/447942\_1/100037358\_1/559015/678560\_1/353247/556638\_1/114457/436768 |
| dre01212 | Fatty acid metabolism | 8/900 | 91/8379 | 0.7753428 | 0.9807608 | 140615/334217/393441/559403\_1/554145/559001\_1/393657/317738\_1 | fads2/elovl7a/hacd3/acaca/elovl8b/fasn/zgc:66024/elovl6 | dre:140615/dre:334217/dre:393441/dre:559403/dre:554145/dre:559001/dre:393657/dre:317738 | 8 | 5 | 140615/393441/559403\_1/554145/393657 | 3 | 334217/559001\_1/317738\_1 |
| dre00650 | Butanoate metabolism | 2/900 | 26/8379 | 0.7853032 | 0.9807608 | 797311/565235\_1 | LOC797311/aldh5a1 | dre:797311/dre:565235 | 2 | 2 | 797311/565235\_1 | 0 |  |
| dre03008 | Ribosome biogenesis in eukaryotes | 7/900 | 83/8379 | 0.8021237 | 0.9807608 | 393107/407084/393107\_2/407084\_1/393107\_1/492761\_1/553787\_1 | utp6/riok2/utp6/riok2/utp6/tbl3/pop4 | dre:393107/dre:407084/dre:393107/dre:407084/dre:393107/dre:492761/dre:553787 | 7 | 5 | 393107\_2/407084\_1/393107\_1/492761\_1/553787\_1 | 2 | 393107/407084 |
| dre00531 | Glycosaminoglycan degradation | 2/900 | 27/8379 | 0.8028278 | 0.9807608 | 565246/567720 | si:dkey-192p21.6/idua | dre:565246/dre:567720 | 2 | 1 | 567720 | 1 | 565246 |
| dre04110 | Cell cycle | 21/900 | 229/8379 | 0.8109296 | 0.9807608 | 560751\_1/431763\_1/431763/566841/323299\_2/323883/327226/767751\_1/554509\_1/novel.902/797406\_2/563708/100141368\_1/324475\_1/562282\_1/794108\_1/562282/393223\_1/327060/280649\_1/559723 | stag2b/gadd45ab/gadd45ab/crebbpa/atrx/cul1a/casc5/ccnb3/orc1/-/espl1/skp2/waplb/smc3/ppp2r5b/nipblb/ppp2r5b/fzr1a/wee1/plk1/tgfb2l | dre:560751/dre:431763/dre:431763/dre:566841/dre:323299/dre:323883/dre:327226/dre:767751/dre:554509/dre:323299/dre:797406/dre:563708/dre:100141368/dre:324475/dre:562282/dre:794108/dre:562282/dre:393223/dre:327060/dre:280649/dre:559723 | 21 | 12 | 560751\_1/431763/566841/327226/767751\_1/554509\_1/100141368\_1/794108\_1/562282/327060/280649\_1/559723 | 9 | 431763\_1/323299\_2/323883/novel.902/797406\_2/563708/324475\_1/562282\_1/393223\_1 |
| dre00970 | Aminoacyl-tRNA biosynthesis | 4/900 | 51/8379 | 0.8125468 | 0.9807608 | 324940\_1/394188/101884223/792787 | aars/qars/LOC101884223/aars2 | dre:792787/dre:394188/dre:101884223/dre:792787 | 4 | 2 | 101884223/792787 | 2 | 324940\_1/394188 |
| dre00061 | Fatty acid biosynthesis | 2/900 | 28/8379 | 0.8190697 | 0.9807608 | 559403\_1/559001\_1 | acaca/fasn | dre:559403/dre:559001 | 2 | 1 | 559403\_1 | 1 | 559001\_1 |
| dre00520 | Amino sugar and nucleotide sugar metabolism | 6/900 | 74/8379 | 0.8206589 | 0.9807608 | 406339\_1/100007686/100885772/563138/321224/751668 | hk2/LOC100007686/cmasb/si:ch211-219a15.4/hkdc1/gck | dre:406339/dre:100007686/dre:100885772/dre:563138/dre:321224/dre:751668 | 6 | 3 | 100885772/321224/751668 | 3 | 406339\_1/100007686/563138 |
| dre00640 | Propanoate metabolism | 3/900 | 41/8379 | 0.8318932 | 0.9807608 | 437019\_1/317746/559403\_1 | pcca/suclg2/acaca | dre:437019/dre:317746/dre:559403 | 3 | 3 | 437019\_1/317746/559403\_1 | 0 |  |
| dre03010 | Ribosome | 14/900 | 161/8379 | 0.8351168 | 0.9807608 | 336712\_2/415196\_1/327089/550564\_1/768187\_1/553284/336712/554098\_2/553284\_1/450009\_1/560828\_1/553799/337007/337007\_1 | rpl10/rpl5b/rpl12/faub/mrpl18/mrpl1/rpl10/mrpl28/mrpl1/mrps16/rpl13a/mrpl30/rps12/rps12 | dre:336712/dre:415196/dre:327089/dre:550564/dre:768187/dre:553284/dre:336712/dre:554098/dre:553284/dre:450009/dre:560828/dre:553799/dre:337007/dre:337007 | 14 | 6 | 336712\_2/415196\_1/327089/553284\_1/560828\_1/337007\_1 | 8 | 550564\_1/768187\_1/553284/336712/554098\_2/450009\_1/553799/337007 |
| dre04114 | Oocyte meiosis | 16/900 | 182/8379 | 0.8364456 | 0.9807608 | 565241\_2/336121/562552/323883/568726/797406\_2/245702\_1/324475\_1/562282\_1/562282/565241/560807/245702/100009657\_1/100005148\_1/280649\_1 | ppp3ccb/calm2a/zgc:171775/cul1a/adcy7/espl1/igf1rb/smc3/ppp2r5b/ppp2r5b/ppp3ccb/adcy6b/igf1rb/rps6ka3b/ar/plk1 | dre:565241/dre:192322/dre:562552/dre:323883/dre:568726/dre:797406/dre:245702/dre:324475/dre:562282/dre:562282/dre:565241/dre:560807/dre:245702/dre:100009657/dre:100005148/dre:280649 | 16 | 10 | 336121/562552/568726/562282/565241/560807/245702/100009657\_1/100005148\_1/280649\_1 | 6 | 565241\_2/323883/797406\_2/245702\_1/324475\_1/562282\_1 |
| dre03450 | Non-homologous end-joining | 1/900 | 16/8379 | 0.8379414 | 0.9807608 | 566285\_1 | dclre1c | dre:566285 | 1 | 1 | 566285\_1 | 0 |  |
| dre04218 | Cellular senescence | 21/900 | 234/8379 | 0.8396311 | 0.9807608 | 333941\_1/565241\_2/431763\_1/336121/393186\_1/431763/562552/100000252\_1/767751\_1/100000252\_3/393176\_1/393176/557551\_1/557551/100330617\_1/100037341/565241/100002946/100000252/572042/559723 | zfp36l2/ppp3ccb/gadd45ab/calm2a/zgc:56235/gadd45ab/zgc:171775/pik3r3b/ccnb3/pik3r3b/rad9a/rad9a/hipk2/hipk2/tsc1b/zgc:162895/ppp3ccb/cxcl8a/pik3r3b/hipk1a/tgfb2l | dre:333941/dre:565241/dre:431763/dre:192322/dre:393186/dre:431763/dre:562552/dre:100000252/dre:767751/dre:100000252/dre:393176/dre:393176/dre:557551/dre:557551/dre:100330617/dre:100037341/dre:565241/dre:100002946/dre:100000252/dre:572042/dre:559723 | 21 | 13 | 333941\_1/336121/393186\_1/431763/562552/767751\_1/100000252\_3/393176/557551\_1/100330617\_1/565241/100000252/559723 | 8 | 565241\_2/431763\_1/100000252\_1/393176\_1/557551/100037341/100002946/572042 |
| dre03050 | Proteasome | 5/900 | 65/8379 | 0.8416938 | 0.9807608 | 30387/796450\_1/326884\_1/796450/436816 | psmb5/adrm1/psmc4/adrm1/zgc:92791 | dre:30387/dre:796450/dre:326884/dre:796450/dre:436816 | 5 | 4 | 30387/796450\_1/326884\_1/436816 | 1 | 796450 |
| dre03040 | Spliceosome | 14/900 | 164/8379 | 0.8538998 | 0.9807608 | 100004402\_1/100037380\_3/573376/100004402/100005893/100005893\_1/566017/100037380\_2/560210/394058\_1/566017\_1/406288\_1/334283\_2/286774\_1 | plrg1/zgc:163098/hspa8/plrg1/magoh/magoh/lsm8/zgc:163098/hsp70l/fus/lsm8/srsf1a/ddx23/phf5a | dre:100004402/dre:100037380/dre:573376/dre:100004402/dre:100005893/dre:100005893/dre:566017/dre:100037380/dre:30671/dre:394058/dre:566017/dre:406288/dre:334283/dre:286774 | 14 | 6 | 100004402\_1/100037380\_3/573376/100005893/566017\_1/334283\_2 | 8 | 100004402/100005893\_1/566017/100037380\_2/560210/394058\_1/406288\_1/286774\_1 |
| dre00600 | Sphingolipid metabolism | 7/900 | 89/8379 | 0.8557938 | 0.9807608 | 445250/445250\_1/450083/100330155/100000684\_1/553981\_1/493602 | neu3.2/neu3.2/gla/cers4b/neu3.5/sptlc1/asah2 | dre:445250/dre:445250/dre:450083/dre:100330155/dre:100000684/dre:553981/dre:493602 | 7 | 2 | 450083/493602 | 5 | 445250/445250\_1/100330155/100000684\_1/553981\_1 |
| dre00532 | Glycosaminoglycan biosynthesis - chondroitin sulfate / dermatan sulfate | 2/900 | 31/8379 | 0.8608244 | 0.9807608 | 100331876/100538167 | LOC100331876/si:dkey-26i13.6 | dre:100331876/dre:100538167 | 2 | 2 | 100331876/100538167 | 0 |  |
| dre00140 | Steroid hormone biosynthesis | 7/900 | 91/8379 | 0.8708207 | 0.9807608 | 406731/393293/553969/100415796/570913\_1/767756/791578\_1 | ugt1ab/hsd11b1la/cyp3a65/ugt5b5/ugt5g1/ugt5a2/dhrs11a | dre:406731/dre:100006793/dre:553969/dre:678597/dre:570913/dre:767756/dre:791578 | 7 | 2 | 553969/791578\_1 | 5 | 406731/393293/100415796/570913\_1/767756 |
| dre04141 | Protein processing in endoplasmic reticulum | 21/900 | 241/8379 | 0.8742198 | 0.9807608 | 327197/386590\_1/386590/327197\_1/266757/266757\_1/573376/337190/567029\_1/566876/323883/556410\_1/565338\_1/567021/108181182/560210/564295\_2/100005358/559564/574002/393877 | vcp/hsp90b1/hsp90b1/vcp/tram1/tram1/hspa8/ssr3/rrbp1b/wfs1a/cul1a/atf4b/eif2ak1/ern2/LOC108181182/hsp70l/traf2a/si:ch211-274f20.2/eif2ak3/zgc:114174/derl3 | dre:327197/dre:386590/dre:386590/dre:327197/dre:266757/dre:266757/dre:573376/dre:337190/dre:567029/dre:566876/dre:323883/dre:556410/dre:565338/dre:567021/dre:567029/dre:30671/dre:564295/dre:100005358/dre:559564/dre:574002/dre:393877 | 21 | 11 | 386590\_1/327197\_1/266757/573376/337190/567029\_1/566876/556410\_1/567021/108181182/574002 | 10 | 327197/386590/266757\_1/323883/565338\_1/560210/564295\_2/100005358/559564/393877 |
| dre04744 | Phototransduction | 4/900 | 57/8379 | 0.8747528 | 0.9807608 | 336121/100330921/792319/140425 | calm2a/si:ch211-103a14.5/saga/gc2 | dre:192322/dre:100330921/dre:792319/dre:140425 | 4 | 3 | 336121/792319/140425 | 1 | 100330921 |
| dre04144 | Endocytosis | 38/900 | 417/8379 | 0.8833170 | 0.9807608 | 474327\_1/336426\_1/793158/792229\_1/573376/436718\_1/792229/336426/406837\_1/406684/792828/100003902\_1/436718/550542\_1/406837/793158\_1/336368/386856\_1/393881\_1/406684\_1/387529/792828\_1/393881/560210/474327/novel.946/571431/245702\_1/568170\_2/100151129/556226\_1/556277/565743\_1/492487/568170\_1/445305\_1/561771/245702 | capza1b/chmp1b/cdc42l/sh3glb1a/hspa8/rhocb/sh3glb1a/chmp1b/rnf41/vps26b/pard6a/si:dkey-16l2.16/rhocb/chmp4c/rnf41/cdc42l/vps37a/pdgfra/vps26a/vps26b/ldlra/pard6a/vps26a/hsp70l/capza1b/-/LOC571431/igf1rb/eps15l1a/rab11fip4b/cdc42l2/cblb/pld2/rab11a/eps15l1a/arpc4l/acap1/igf1rb | dre:474327/dre:336426/dre:793158/dre:792229/dre:573376/dre:436718/dre:792229/dre:336426/dre:406837/dre:406684/dre:792828/dre:100003902/dre:436718/dre:550542/dre:406837/dre:793158/dre:336368/dre:386856/dre:393881/dre:406684/dre:387529/dre:792828/dre:393881/dre:30671/dre:474327/dre:386856/dre:571431/dre:245702/dre:568170/dre:100151129/dre:556226/dre:556277/dre:565743/dre:492487/dre:568170/dre:445305/dre:561771/dre:245702 | 38 | 17 | 792229\_1/573376/336426/406837\_1/436718/793158\_1/336368/406684\_1/792828\_1/393881/474327/556226\_1/556277/565743\_1/568170\_1/561771/245702 | 21 | 474327\_1/336426\_1/793158/436718\_1/792229/406684/792828/100003902\_1/550542\_1/406837/386856\_1/393881\_1/387529/560210/novel.946/571431/245702\_1/568170\_2/100151129/492487/445305\_1 |
| dre04012 | ErbB signaling pathway | 10/900 | 127/8379 | 0.8881371 | 0.9807608 | 100000252\_1/544664/544664\_1/100000252\_3/100006675/571431/556277/58100\_1/100000252/497384\_1 | pik3r3b/erbb3b/erbb3b/pik3r3b/prkcba/LOC571431/cblb/badb/pik3r3b/prkca | dre:100000252/dre:544664/dre:100149258/dre:100000252/dre:100006675/dre:571431/dre:556277/dre:58100/dre:100000252/dre:497384 | 10 | 5 | 544664\_1/100000252\_3/100006675/556277/100000252 | 5 | 100000252\_1/544664/571431/58100\_1/497384\_1 |
| dre04512 | ECM-receptor interaction | 10/900 | 128/8379 | 0.8935642 | 0.9807608 | 563428/570868\_1/563428\_1/321243\_1/30037/798998/322012\_1/100005469\_1/797491\_1/564266 | fras1/itga1/fras1/lama5/tnc/gp1bb/hmmr/fn1a/itgb7/itgb3a | dre:563428/dre:570868/dre:563428/dre:321243/dre:30037/dre:798998/dre:322012/dre:100005469/dre:797491/dre:564266 | 10 | 8 | 570868\_1/563428\_1/321243\_1/30037/798998/322012\_1/100005469\_1/564266 | 2 | 563428/797491\_1 |
| dre03250 | Viral life cycle - HIV-1 | 6/900 | 83/8379 | 0.8938579 | 0.9807608 | 566841/64275\_1/559677\_1/321602\_1/562808\_1/559032\_1 | crebbpa/supt5h/nelfa/cdk9/map1sa/map1b | dre:566841/dre:64275/dre:559677/dre:321602/dre:562808/dre:559032 | 6 | 2 | 566841/64275\_1 | 4 | 559677\_1/321602\_1/562808\_1/559032\_1 |
| dre00534 | Glycosaminoglycan biosynthesis - heparan sulfate / heparin | 2/900 | 38/8379 | 0.9261766 | 0.9914116 | 792524/791107\_1 | hs6st3a/hs6st1b | dre:792524/dre:791107 | 2 | 2 | 792524/791107\_1 | 0 |  |
| dre00430 | Taurine and hypotaurine metabolism | 1/900 | 23/8379 | 0.9269871 | 0.9914116 | 794311 | fmo5 | dre:794311 | 1 | 1 | 794311 | 0 |  |
| dre00760 | Nicotinate and nicotinamide metabolism | 3/900 | 52/8379 | 0.9287530 | 0.9914116 | 402953\_1/101886891/110438097 | pnp6/LOC101886891/LOC110438097 | dre:402953/dre:101886891/dre:572070 | 3 | 3 | 402953\_1/101886891/110438097 | 0 |  |
| dre00515 | Mannose type O-glycan biosynthesis | 2/900 | 39/8379 | 0.9327113 | 0.9914116 | 569769/100093713 | pomt1/zgc:165519 | dre:569769/dre:100093713 | 2 | 0 |  | 2 | 569769/100093713 |
| dre04371 | Apelin signaling pathway | 18/900 | 226/8379 | 0.9349388 | 0.9914116 | 323509\_1/117508\_1/336121/30498\_1/117508/30498/321449/100331581/568726/677750/100331581\_1/335655/100037358\_1/321449\_1/560807/557486\_1/335655\_1/557486 | gnaia/klf2a/calm2a/egr1/klf2a/egr1/ctgfa/si:dkey-44g17.6/adcy7/myl2b/si:dkey-44g17.6/gngt2a/zgc:163073/ctgfa/adcy6b/hdac5/gngt2a/hdac5 | dre:323509/dre:117508/dre:192322/dre:30498/dre:117508/dre:30498/dre:321449/dre:100331581/dre:568726/dre:677750/dre:100331581/dre:335655/dre:100037358/dre:321449/dre:560807/dre:557486/dre:335655/dre:557486 | 18 | 12 | 323509\_1/117508\_1/336121/30498\_1/117508/321449/100331581/568726/677750/560807/557486\_1/335655\_1 | 6 | 30498/100331581\_1/335655/100037358\_1/321449\_1/557486 |
| dre00230 | Purine metabolism | 14/900 | 192/8379 | 0.9595779 | 0.9995734 | 100330864/334431\_1/560827\_1/777719/568726/550560\_1/553639\_1/402953\_1/333997/560807/100005067\_1/447905/140425/333997\_1 | LOC100330864/adsl/prps1b/papss2a/adcy7/entpd6/gmpr/pnp6/ampd3b/adcy6b/dguok/entpd2a.1/gc2/ampd3b | dre:100330864/dre:334431/dre:560827/dre:777719/dre:568726/dre:550560/dre:553639/dre:402953/dre:333997/dre:560807/dre:100005067/dre:447905/dre:140425/dre:333997 | 14 | 12 | 100330864/334431\_1/560827\_1/568726/550560\_1/553639\_1/402953\_1/333997/560807/447905/140425/333997\_1 | 2 | 777719/100005067\_1 |
| dre00190 | Oxidative phosphorylation | 12/900 | 171/8379 | 0.9632656 | 0.9995734 | 393793\_1/447914\_1/573619/359840\_1/447942\_1/359840/767750\_2/447942/767750/796997\_1/359839/436768 | uqcrc1/cox17/ndufab1a/atp6v1b2/cox6a2/atp6v1b2/atp5ib/cox6a2/atp5ib/ndufs7/atp6v1ba/uqcrq | dre:393793/dre:447914/dre:573619/dre:359840/dre:447942/dre:359840/dre:767750/dre:447942/dre:767750/dre:796997/dre:359839/dre:436768 | 12 | 7 | 447914\_1/573619/359840/767750\_2/447942/767750/359839 | 5 | 393793\_1/359840\_1/447942\_1/796997\_1/436768 |
| dre04814 | Motor proteins | 23/900 | 305/8379 | 0.9776644 | 0.9995734 | 57934\_1/337226\_1/474327\_1/337226/57934/30429\_2/334274/100149148/798632/406811\_1/101882034/474327/677750/100037358\_1/556023\_1/799461\_2/353247/431777/556023/30429/571487/562052\_1/100334351 | actb1/zgc:153867/capza1b/zgc:153867/actb1/mylpfa/myhc4/myha/myl6/zgc:55461/LOC101882034/capza1b/myl2b/zgc:163073/dnah2/dnah12/tnnc1a/tuba7l/dnah2/mylpfa/si:dkeyp-92c9.3/kif14/dnah3 | dre:57934/dre:337226/dre:474327/dre:337226/dre:57934/dre:30429/dre:334274/dre:100149148/dre:798632/dre:336681/dre:406297/dre:474327/dre:677750/dre:100037358/dre:556023/dre:799461/dre:353247/dre:431777/dre:556023/dre:30429/dre:571487/dre:562052/dre:100334351 | 23 | 13 | 57934\_1/337226\_1/798632/101882034/474327/677750/556023\_1/799461\_2/431777/30429/571487/562052\_1/100334351 | 10 | 474327\_1/337226/57934/30429\_2/334274/100149148/406811\_1/100037358\_1/353247/556023 |
| dre00220 | Arginine biosynthesis | 1/900 | 35/8379 | 0.9814187 | 0.9995734 | 406688 | got2a | dre:406688 | 1 | 0 |  | 1 | 406688 |
| dre00601 | Glycosphingolipid biosynthesis - lacto and neolacto series | 1/900 | 42/8379 | 0.9916446 | 0.9995734 | 100093713 | zgc:165519 | dre:100093713 | 1 | 0 |  | 1 | 100093713 |
| dre04340 | Hedgehog signaling pathway | 3/900 | 79/8379 | 0.9932211 | 0.9995734 | 323883/30269\_1/100006858\_1 | cul1a/shha/csnk1e | dre:323883/dre:30269/dre:100006858 | 3 | 0 |  | 3 | 323883/30269\_1/100006858\_1 |
| dre04350 | TGF-beta signaling pathway | 8/900 | 161/8379 | 0.9971059 | 0.9995734 | 436718\_1/566841/436718/323883/100004116\_1/405778/556634/559723 | rhocb/crebbpa/rhocb/cul1a/fsta/grem1a/adamtsl7/tgfb2l | dre:436718/dre:566841/dre:436718/dre:323883/dre:100004116/dre:100332300/dre:556634/dre:559723 | 8 | 5 | 566841/436718/100004116\_1/405778/559723 | 3 | 436718\_1/323883/556634 |
| dre04920 | Adipocytokine signaling pathway | 4/900 | 108/8379 | 0.9979754 | 0.9995734 | 406473/100037357/564295\_2/100149042 | pck2/tnfrsf1b/traf2a/agrp | dre:406473/dre:100037357/dre:564295/dre:100149042 | 4 | 2 | 406473/100149042 | 2 | 100037357/564295\_2 |
| dre00510 | N-Glycan biosynthesis | 1/900 | 68/8379 | 0.9995734 | 0.9995734 | 327601\_1 | alg8 | dre:327601 | 1 | 0 |  | 1 | 327601\_1 |
